# Which Way In? The RalF Arf-GEF Orchestrates *Rickettsia* Host Cell Invasion

**DOI:** 10.1371/journal.ppat.1005115

**Published:** 2015-08-20

**Authors:** Kristen E. Rennoll-Bankert, M. Sayeedur Rahman, Joseph J. Gillespie, Mark L. Guillotte, Simran J. Kaur, Stephanie S. Lehman, Magda Beier-Sexton, Abdu F. Azad

**Affiliations:** Department of Microbiology and Immunology, University of Maryland School of Medicine, Baltimore, Maryland, United States of America; Johns Hopkins School of Public Health, UNITED STATES

## Abstract

Bacterial Sec7-domain-containing proteins (RalF) are known only from species of *Legionella* and *Rickettsia*, which have facultative and obligate intracellular lifestyles, respectively. *L*. *pneumophila* RalF, a type IV secretion system (T4SS) effector, is a guanine nucleotide exchange factor (GEF) of ADP-ribosylation factors (Arfs), activating and recruiting host Arf1 to the *Legionella*-containing vacuole. In contrast, previous *in vitro* studies showed *R*. *prowazekii* (Typhus Group) RalF is a functional Arf-GEF that localizes to the host plasma membrane and interacts with the actin cytoskeleton via a unique C-terminal domain. As RalF is differentially encoded across *Rickettsia* species (e.g., pseudogenized in all Spotted Fever Group species), it may function in lineage-specific biology and pathogenicity. Herein, we demonstrate RalF of *R*. *typhi* (Typhus Group) interacts with the *Rickettsia* T4SS coupling protein (RvhD4) via its proximal C-terminal sequence. RalF is expressed early during infection, with its inactivation via antibody blocking significantly reducing *R*. *typhi* host cell invasion. For *R*. *typhi* and *R*. *felis* (Transitional Group), RalF ectopic expression revealed subcellular localization with the host plasma membrane and actin cytoskeleton. Remarkably, *R*. *bellii* (Ancestral Group) RalF showed perinuclear localization reminiscent of ectopically expressed *Legionella* RalF, for which it shares several structural features. For *R*. *typhi*, RalF co-localization with Arf6 and PI(4,5)P_2_ at entry foci on the host plasma membrane was determined to be critical for invasion. Thus, we propose recruitment of PI(4,5)P_2_ at entry foci, mediated by RalF activation of Arf6, initiates actin remodeling and ultimately facilitates bacterial invasion. Collectively, our characterization of RalF as an invasin suggests that, despite carrying a similar Arf-GEF unknown from other bacteria, different intracellular lifestyles across *Rickettsia* and *Legionella* species have driven divergent roles for RalF during infection. Furthermore, our identification of lineage-specific Arf-GEF utilization across some rickettsial species illustrates different pathogenicity factors that define diverse agents of rickettsial diseases.

## Author Summary

Phylogenomics analysis indicates divergent mechanisms for host cell invasion across diverse species of obligate intracellular *Rickettsia*. For instance, only some *Rickettsia* species carry RalF, the rare bacterial Arf-GEF effector utilized by *Legionella pneumophila* to facilitate fusion of ER-derived membranes with its host-derived vacuole. For *R*. *prowazekii* (Typhus Group, TG), prior *in vitro* studies suggested the Arf-GEF activity of RalF, which is absent from Spotted Fever Group species, might be spatially regulated at the host plasma membrane. Herein, we demonstrate RalF of *R*. *typhi* (TG) and *R*. *felis* (Transitional Group) localizes to the host plasma membrane, yet *R*. *bellii* (Ancestral Group) RalF shows perinuclear localization reminiscent of RalF-mediated recruitment of Arf1 by *L*. *pneumophila* to its vacuole. For *R*. *typhi*, RalF expression occurs early during infection, with RalF inactivation significantly reducing host cell invasion. Furthermore, RalF co-localization with Arf6 and the phosphoinositide PI(4,5)P_2_ at the host plasma membrane was determined to be critical for *R*. *typhi* invasion. Thus, our work illustrates that different intracellular lifestyles across species of *Rickettsia* and *Legionella* have driven divergent roles for RalF during host cell infection. Collectively, we identify lineage-specific Arf-GEF utilization across diverse rickettsial species, previously unappreciated mechanisms for host cell invasion and infection.

## Introduction

Bacteria invading eukaryotic cells employ diverse strategies for successful entry, intracellular colonization and intercellular spread [1,2]. Whether facultative or obligate, intracellular species must either modify the phagocytic vacuole for survival or lyse the phagosome and live freely within the host cytoplasm (or invade other cellular organelles) [3–6]. Either strategy is delicately underpinned by bacterial secretion of effectors, which have a myriad of characterized functions: e.g., engaging host signaling pathways, rearranging the host cytoskeleton, polymerizing host actin, subverting host vesicular traffic, etc. [7–9]. It is well established that divergent effectors from distantly-related intracellular species can operate in similar processes [10]; e.g., actin nucleators from species of *Shigella*, *Listeria* and *Rickettsia* [11,12] and phospholipases from species of *Pseudomonas* and *Legionella* [13,14]. Conversely, the ability for highly similar effectors from distantly-related species to function differently in host cells is a phenomenon that is poorly known, probably reflective of effector repertoires being highly specific to bacterial genera [15–17].

Species of *Rickettsia* (*Alphaproteobacteria*: Rickettsiales) are Gram-negative obligate intracellular parasites of a wide range of eukaryotic species [18]. Rickettsiae bind to host cells and induce phagocytosis [19,20], with internalized bacteria released into the cytosol upon rapid escape from the phagocytic vacuole. Bacteria spread intercellularly upon death and lysis of host cells, though some species move intercellularly prior to host cell lysis via host actin polymerization [21–23]. Several surface proteins characterized for adhesion and/or entry of host cells (Sca5, Adr1, Adr2) [24–28] and activation of cytoskeletal vinculin (Sca4) [29] are conserved across sequenced *Rickettsia* genomes, as are several enzymes implicated in phagosomal lysis (TlyC, PLD, Pat1) [30–33]. In contrast, other characterized adhesins (Sca0, Sca1, Sca2) [34–38], proteins involved in Arp2/3-dependent (RickA) [39,40] and -independent (Sca2) [41,42] host actin polymerization, and another phospholipase (Pat2) [43,44] are sporadically encoded across rickettsial lineages. This suggests that, despite superficially similar infection strategies, diverse *Rickettsia* species employ distinct molecular mechanisms for successful colonization of host cells [45].

One such protein that is differentially encoded across *Rickettsia* genomes is a highly similar counterpart to the RalF protein of *Legionella* spp. Collectively, these proteins contain a Sec7-domain, which in eukaryotes functions as a guanine nucleotide exchange factor (GEF) of ADP-ribosylation factors (Arfs) [46]. Remarkably, bacterial Sec7-domain containing proteins are unknown from other bacteria [47]. *Legionella* RalF (RalF_L_) is a secreted effector, with its proximal C-terminal sequence mediating secretion through the *dot*/*icm* type IV secretion system (T4SS) [48]. RalF_L_ activates and recruits host Arf1 to the *Legionella*-containing vacuole (LCV), which is a modification of the phagosome [49]. The structure of RalF_L_ contains two distinct domains: an N-terminal Sec7 domain (S7D) and a C-terminal Sec7-capping domain (SCD) that regulates active site access to Arfs [50]. The S7D and SCD across RalF_L_ and *Rickettsia* RalF (RalF_R_) share ~45% aa identity, though an extended variable region flanks the SCD of RalF_R_ proteins at the C-terminus [51].

A comparative study of RalF_L_ and RalF_R_ determined similar GEF activities for both proteins, yet divergent subcellular localization patterns driven primarily by intrinsic characteristics of the SCD [52]. The RalF_L_ SCD positions the protein at the endoplasmic reticulum for interception of host secretory vesicles, while the RalF_R_ SCD targets the protein to the host plasma membrane. Furthermore, a proline-rich region within the extended variable region of RalF_R_ interacts with components of the host actin cytoskeleton. Subsequently, membrane sensor regions were identified within the SCDs of RalF_L_ and RalF_R_, with differential enrichments in aromatic and positively charged residues determining divergent lipid substrates that regulate Arf-GEF activities [53]. Collectively, these studies suggest that these distinguishing features (divergent SCD sensor regions, RalF_R_-specific cytoskeletal-binding domain) mediate the spatial regulation of RalF activity in two diverse intracellular species with very different lifestyles.

Despite tremendous insight on the possible function of RalF_R_ during rickettsial host cell infection, important questions are left unanswered. As previous studies were performed *in vitro* [52,53], it still remains unknown if those *Rickettsia* species that carry *ralF* genes actually express RalF_R_ during infection, and if so, at what time point. Furthermore, as GEFs confer the spatial regulation of different Arf classes at discrete cellular locales [54–57], the Arf(s) specificity of RalF_R_ needs to be determined in light of the different subcellular localization of the protein compared to *Legionella* spp. Our work presented here addresses these unknowns by demonstrating RalF expression by *R*. *typhi* early during host cell invasion. Across several *Rickettsia* species, we identified the domain requirements for positioning RalF at host membranes, and for *R*. *typhi*, determined that RalF co-localization with Arf6 and PI(4,5)P_2_ at entry foci was critical for invasion. Altogether, our work identifies Arf-GEF utilization as a lineage-specific invasion mechanism, illuminating the variable strategies that drive *Rickettsia* infection of host cells.

## Results

### RalF_Rt_ interacts with the *rvh* T4SS coupling protein (RvhD4) via its proximal C-terminal sequence and is secreted during host cell infection

**Fig 1:**
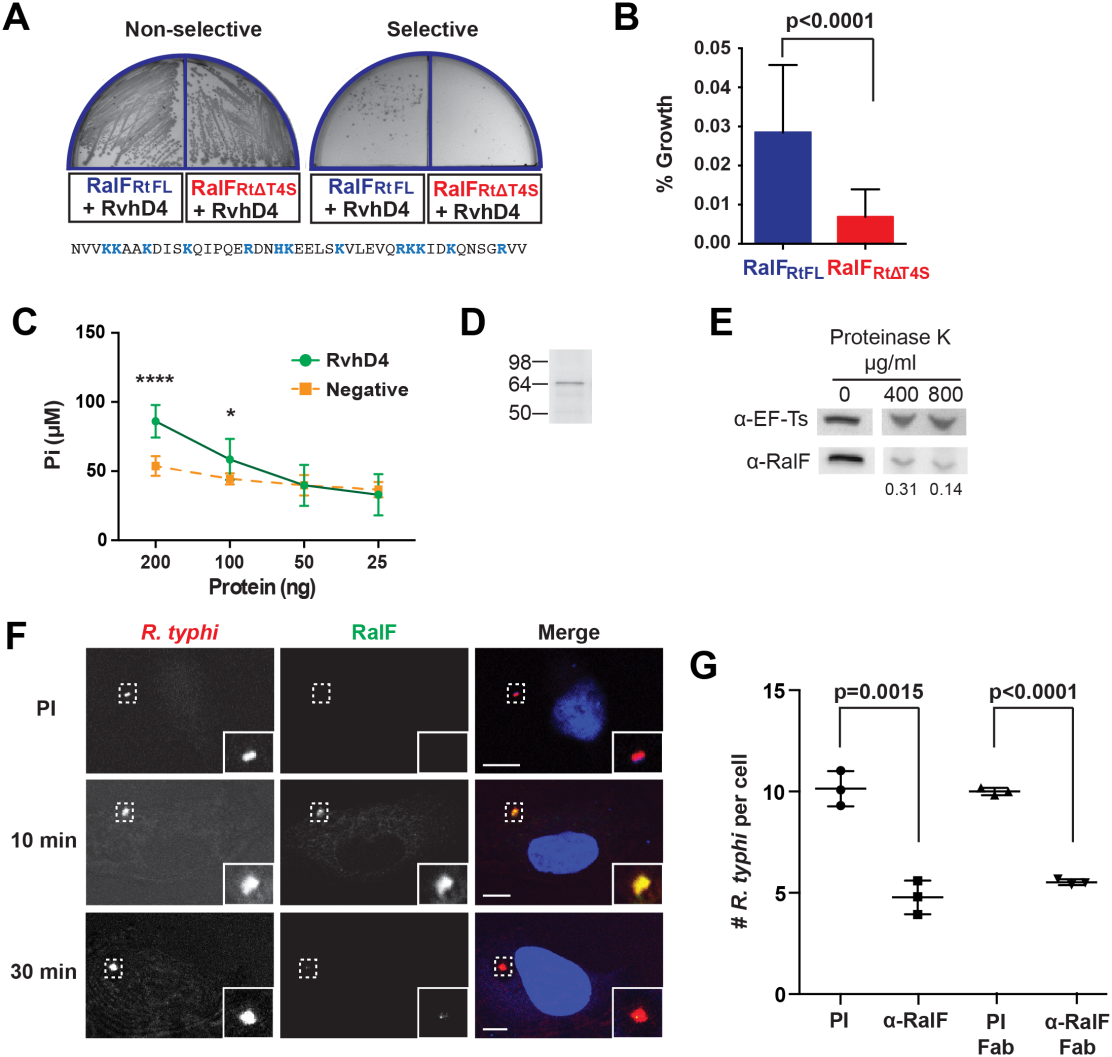
*R*. *typhi* RalF_Rt_ interacts with RvhD4 and is expressed early during host cell invasion. (A) Bacterial two-hybrid (B2H) assay reveals an interaction between RalF_Rt_ and RvhD4. *ralF*_*RtFL*_ and *ralF*_*RtΔT4S*_ were cloned into pTRG (prey) and *rvhD4* was cloned into pBT (bait) of the B2H system. Constructed bait and prey plasmids were co-transformed into BacterioMatch II reporter electro-competent cells. Transformants were screened on non-selective plate (left) and positive interactions were identified on dual selective screening plate (right). The amino acid sequence deleted from *ralF*_RtΔT4S_ (positively charged residues are colored blue) is shown at bottom. (B) Quantification of bacterial growth in the B2H assay described in panel A. Percent growth of CFUs of reporter cells harboring recombinant plasmids on dual selective screening medium was calculated relative to CFUs obtained on non-selective medium. Error bars represent mean ± SD of three independent experiments (Student’s two-sided t-test). (C) *R*. *typhi* RvhD4 exhibits ATPase activity. A series dilution of purified RvhD4 in assay buffer was incubated with reagent for 30 min at 21°C. The inorganic phosphate (Pi) released from ATP was quantified by measuring absorbance at OD 620 nm. As a negative control, a non-related *R*. *typhi* protein (RT0600) was assayed. Error bars represent mean ± SD of three independent experiments. * p = 0.01, **** p<0.0001; Student’s two-sided t-test. (D) Protein immunoblot of recombinant RvhD4 (~64 kDa) used in ATPase activity assays described in panel C. (E) RalF_Rt_ is surface exposed. Purified *R*. *typhi* was treated with 400 μg/mL or 800 μg/mL Proteinase K or in buffer alone for 1 hr. Lysates were resolved and immunoblotted for RalF or the *R*. *typhi* cytoplasmic control protein, elongation factor Ts (EF-Ts). Densitometry was performed using ImageJ and the intensity of RalF was normalized to EF-Ts. Representative image from two independent experiments is shown. Intensity of RalF normalized to EF-Ts and relative to untreated control is shown below the immunoblots. (F) RalF is expressed during early infection. HeLa cells infected with *R*. *typhi* for 10 and 30 min were fixed and *R*. *typhi* and RalF detected with rat anti-*R*. *typhi* (red) and affinity purified rabbit anti-RalF_Rt_ (green) antibodies, respectively. DAPI (blue) is shown in the merged image. Boxed regions are enlarged to show detail. Pre-immune (PI) cells were treated with rabbit PI serum in place of anti-RalF_Rt_ antibody. (Scale bar: 10 μm). (G) Anti-RalF_Rt_ IgG and Fab fragments inhibit *R*. *typhi* host cell infection. HeLa cells were infected with partially purified *R*. *typhi* pre-absorbed for 30 min with 20μg PI IgG serum, anti-RalF_Rt_ IgG, PI Fab fragments or anti-RalF_Rt_ Fab fragments. Cells were fixed 2 hrs post infection and *R*. *typhi* and the cell membrane detected with anti-*R*. *typhi* serum and Alexa Fluor 594 wheat germ agglutinin, respectively. The number of *R*. *typhi* per host cell was counted for 100 individual host cells in three independent experiments and normalized to PI serum. Error bars represent mean ± SD (Student’s two-sided t-test).

As predicted Arf-GEFs, we anticipated RalF_R_ proteins to be secreted extracellularly into the host cell. Prior to invasion, *L*. *pneumophila* utilizes its *dot*/*icm* I-T4SS to translocate RalF_L_ into host cells [48]. Like RalF_L_, *R*. *typhi* RalF (RalF_Rt_) lacks a predicted N-terminal Sec secretion signal [58], trans-membrane spanning regions [59] and a β-barrel structure [60], suggesting its secretion via a Sec-independent pathway, possibly the Rickettsiales *vir* homolog (*rvh*) T4SS [61]. Accordingly, in order to determine if RalF_Rt_ interacts with the *rvh* T4SS, we performed a bacterial two-hybrid assay with full length RalF_Rt_ (RalF_RtFL_) and RvhD4, the *rvh* T4SS coupling protein. T4SS coupling proteins (VirD4 family) are ATPases that function as “gatekeepers” to regulate substrate entry into the T4SS channel [62,63]. Co-transformation of bait (encoding RvhD4) and prey (encoding RalF_RtFL_) plasmids in BacterioMatch II reporter electrocompetent cells resulted in bacterial growth on selective media (Fig 1A), indicating RalF_RtFL_ and RvhD4 interact, and thus implicating RalF_Rt_ as an *rvh* T4SS effector.

Secretion of RalF_L_ is dependent on hydrophobic residues within its C-terminal tail [48], while many other T4SS protein substrates have enrichments of positively charged residues at their C-termini that are important for secretion [64–66]. Accordingly, we evaluated RalF_Rt_ for the presence of a T4SS signal sequence (T4S) within its C-terminus. A T4S RalF_Rt_ truncation (RalF_RtΔT4S_) was generated and tested for its ability to bind RvhD4 via the bacterial two-hybrid assay (Fig 1A). The percent growth of colony forming units (CFUs) of reporter cells harboring recombinant plasmids on dual selective screening medium was calculated relative to percent growth of CFUs obtained on non-selective His dropout medium by drop plate method for counting. An approximately 77% decrease in CFUs on dual selective media was observed with RalF_RtΔT4S_ compared to RalF_RtFL_, indicating that the RalF C-terminus is important for interacting with RvhD4 (Fig 1B).

The ATPase activity of T4SS coupling proteins is essential for substrate translocation [67]. To confirm functionality of RvhD4, recombinant RvhD4 was assayed for ATPase activity. RvhD4 was found to release inorganic phosphate (Pi) from ATP in a concentration dependent manner compared to a rickettsial protein that lacks predicted ATPase activity (RT0600) (Fig 1C and 1D). This indicates *Rickettsia* RvhD4 is a functional ATPase that likely regulates protein secretion through the *rvh* T4SS.

The interaction of RalF_Rt_ with machinery of the *rvh* T4SS implies extracellular secretion. Our prior report that characterized the *R*. *typhi* surface proteome demonstrated that RalF_Rt_ is expressed and surface exposed [68]. To further confirm RalF_Rt_ secretion, purified *R*. *typhi* was treated with proteinase K, on the premise that surface exposed protein would be degraded with proteinase K treatment while subsurface proteins would be protected. Protease treatment caused a dose-dependent degradation of RalF_Rt_ with respect to the *R*. *typhi* cytoplasmic control protein, elongation factor Ts (EF-Ts, Fig 1E).

### RalF_Rt_ is expressed early during infection and is required for *R*. *typhi* invasion of host cells

To determine when RalF_Rt_ is expressed during *R*. *typhi* infection, a polyclonal antibody against RalF_Rt_ was generated, qualified (S1 Fig) and used for immunofluorescence assays (Fig 1F). During early infection of host cells (10 min), RalF_Rt_ expression is high and diminishes as internalization progresses (30 min). Given RalF_Rt_ expression during early infection, we assessed its role during *R*. *typhi* invasion of host cells. When *R*. *typhi* was pre-treated with the anti-RalF_Rt_ polyclonal antibody, the average number of *R*. *typhi* per host cell decreased by 52% from an average of 10 to 4.8 bacteria per host cell (Fig 1G), indicating a role for RalF during host cell invasion. To rule out possible steric hindrance induced by the Fc portion of the anti-RalF_Rt_ antibody inhibiting rickettsial-host cell interactions that promote entry, *R*. *typhi* was pre-absorbed with anti-RalF_Rt_ Fab fragments. The average number of *R*. *typhi* per host cell was significantly decreased by 45% from 10 to 5.5 bacteria per host cell (Fig 1G) further confirming the involvement of RalF in host cell invasion.

### RalF_R_ is divergent from RalF_L_ within the SCD lipid sensor region and also contains a C-terminal extension that is highly variable across *Rickettsia* homologs

**Fig 2:**
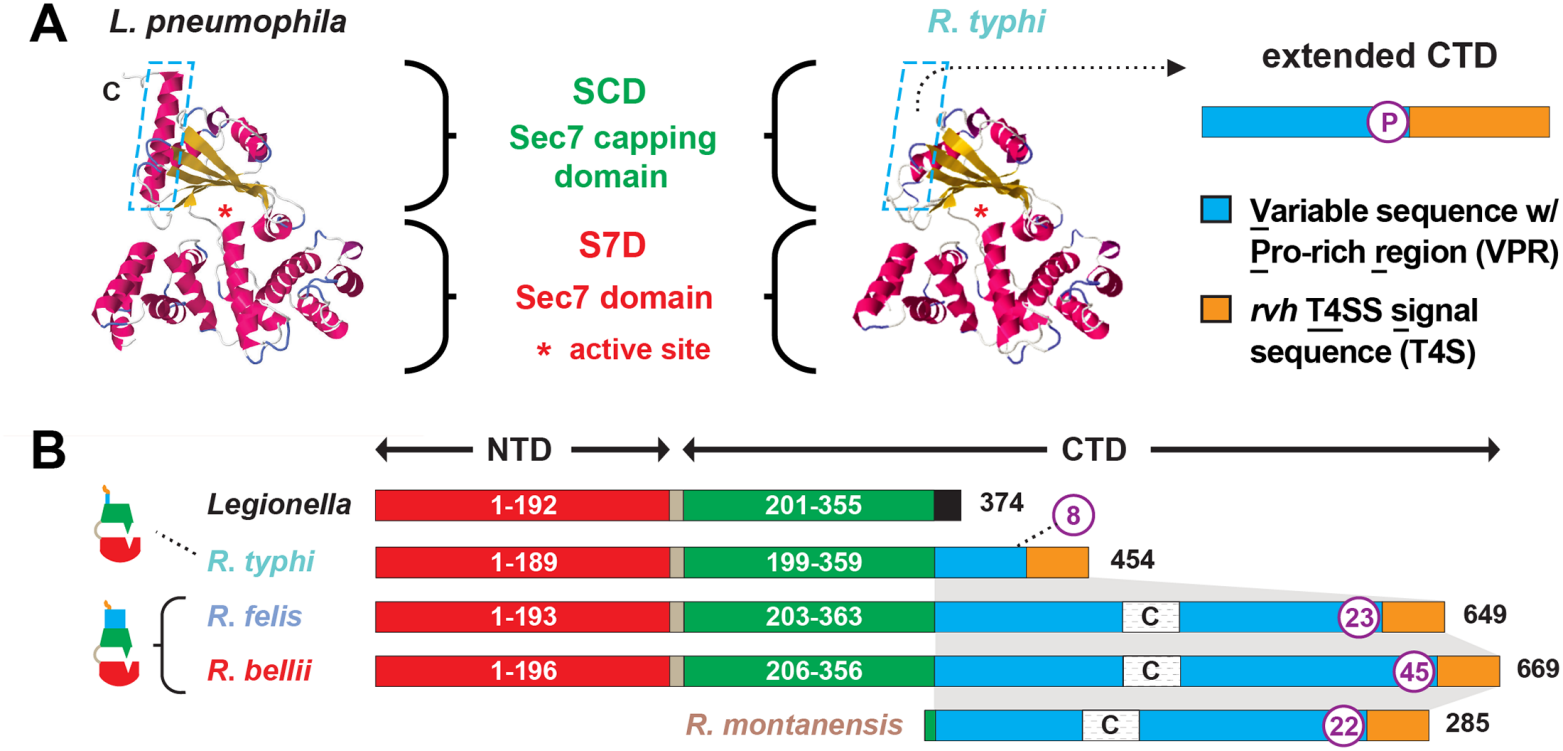
Characteristics and comparative analysis of bacterial Sec7 domain-containing proteins (RalF). (A) Comparison of the crystal structure of *Legionella pneumophila* RalF (PDB 4C7P) [53] with the predicted structure of *R*. *typhi* RalF (RT0362). Modeling done with Phyre2 [69]. The delineation of the Sec7 domain (S7D, red) and Sec7-capping domain (SCD, green) is shown, with an approximation of the active site Glu (asterisk), which is essential for Arf recruitment to the *Legionella* containing vacuole [52]. The distinguishing feature of the otherwise highly similar proteins is the extended C-terminal domain in *R*. *typhi* RalF relative to *L*. *pneumophila* RalF. The blue dashed box depicts the extended C-terminal domain of *Rickettsia* RalF sequences, which can be delineated into a variable sequence with Pro-rich region (VPR) and an *rvh* T4SS signal sequence (T4S). (B) Domain organization of *Legionella* and *Rickettsia* RalF proteins. The structural conservation witnessed in panel A is encoded by conserved S7D (S2B Fig) and SCD (S3B Fig) sequences (~45% ID across *Legionella* and *Rickettsia*). *Rickettsia* RalF VPRs vary extensively across homologs; some *Rickettsia* RalF proteins contain only the VPR and T4S (S4A Fig). C, coiled-coil. Number of Pro residues within purple circles. NCBI GenBank accession numbers for all proteins are provided in the legend of S2 Fig.

Utilizing over 60 *Rickettsia* genome sequences, phylogenomics analyses were carried out to provide further insight on the role of RalF in rickettsial biology and pathogenesis. While a key factor in *R*. *typhi* infection of host cells, RalF-mediated invasion is not a strategy employed by all *Rickettsia* species, as evident by *ralF* pseudogenization in all species of Spotted Fever Group (SFG) rickettsiae, as well as two other species (*R*. *canadensis* and *R*. *helvetica*) [45]. Still, the remaining species, including *R*. *bellii* and all species within the Typhus Group (TG) and Transitional Group (TRG) rickettsiae, contain genes encoding RalF_R_ proteins that are highly conserved within the S7D and SCD as compared to RalF_L_ (Fig 2). Specifically, and in agreement with previous studies [52,53], all RalF_L_ and RalF_R_ proteins contain a highly conserved Sec7 active site within the S7D (S2A and S2B Fig), with RalF_R_ proteins having an enrichment of positively charged residues in the lipid sensor region of the SCD relative to RalF_L_ proteins (S3C Fig). Thus, based on these characteristics, all RalF_R_ proteins are predicted to spatially regulate their Arf-GEF activities at the host plasma membrane, where concentrated negatively charged phospholipids attract the RalF_R_ SCD [53].

Relative to RalF_L_, the major distinguishing factor of RalF_R_ proteins is the presence of a variable sequence with Pro-rich region (VPR) within the C-terminal domain (Fig 2). Pro-rich regions are a common characteristic of proteins that target the actin cytoskeleton [70], and are typically present in Arf-GEFs recruited to cytoskeletal/plasma membrane junctions [71,72]. Across RalF_R_ proteins, the VPR is flanked by the SCD and T4S and is extraordinarily variable in sequence length and number of Pro residues across RalF_R_ proteins (Fig 2B). Remarkably, many SFG rickettsiae species, e.g. *R*. *montanensis*, contain putative ORFs encoding complete VPRs. Alignment of these ORFs with VPRs from full-length RalF_R_ proteins illustrates that a gene encoding RalF_R_ was present in the *Rickettsia* ancestor, with pseudogenization purging the complete Arf-GEF from most *Rickettsia* genomes (S4A Fig). This conclusion is supported by genome synteny analysis across *ralF*_*R*_ loci, which indicates a conserved position for *ralF* flanking the *maeB* gene in all sequenced *Rickettsia* genomes (S5A Fig). Thus, RalF_L_ and RalF_R_ proteins diversified early upon their establishment in ancestral *Legionella* and *Rickettsia* genomes, with the retention of VPRs within full-length RalF_R_ proteins implying an important function.

### The RalF_R_ SCD regulates membrane localization while the VPR targets the host cytoskeleton

**Fig 3:**
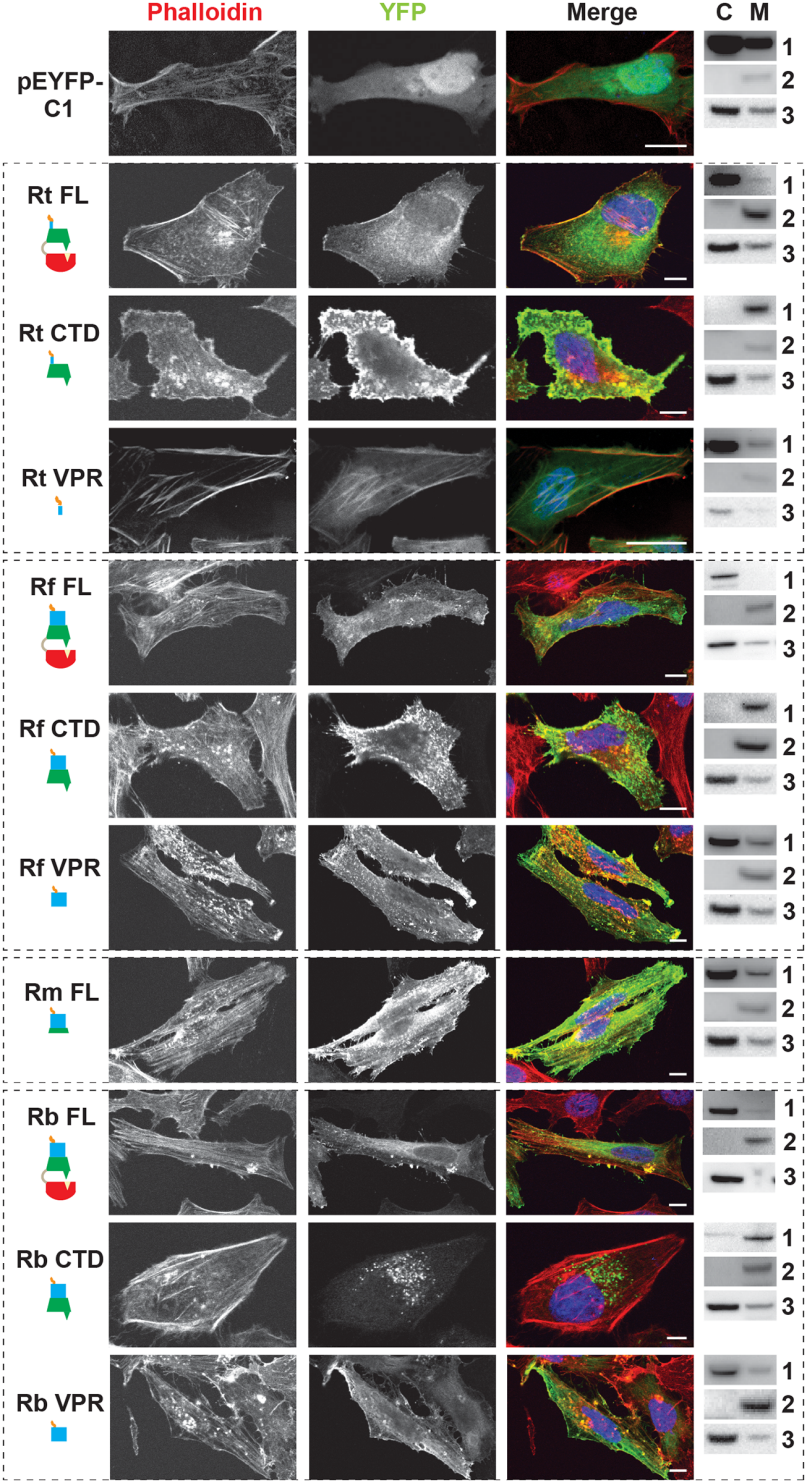
RalF subcellular localization and actin filament disruption mediated by the SCD and VPR. HeLa cells transfected with YFP tagged constructs (green, described in Fig 2B) were stained with Alexa Fluor 594 phalloidin to detect actin (red). DAPI (blue) is shown in the merged image. Cytoplasmic (C) and membrane (M) localization was confirmed via membrane fractionation of HEK293T cells Lipofectamine 2000 transfected with the indicated plasmids followed by immunoblotting. Immunoblot primary antibodies: 1, rabbit anti-GFP (Life Technologies); 2, membrane marker rabbit anti-Calnexin (Abcam); 3, cytoplasmic marker mouse anti-GAPDH (Abcam). Rt, *R*. *typhi*; Rf, *R*. *felis*; Rm, *R*. *montanensis*; Rb, *R*. *bellii*. (Scale bar: 10 μm).

In light of the variability across the VPR of RalF_R_ proteins, we determined the C-terminal domain (CTD) requirements for subcellular localization across RalF_R_ proteins from several species (*R*. *typhi*, *R*. *felis*, *R*. *montanensis* and *R*. *bellii*) (Fig 3). The SCD- and VPR-mediated targeting to the host plasma membrane and actin cytoskeleton, respectively, for RalF of *R*. *prowazekii* (RalF_Rp_) was used as a reference [52,53]. *R*. *typhi* and *R*. *felis* full-length RalF (RalF_FL_) proteins primarily had diffuse staining within the cytoplasm with some plasma membrane localization. However, RalF_CTD_ (SCD-VPR-T4S) localized strongly to the plasma membrane and disrupted actin stress fibers. Additionally, *R*. *typhi* and *R*. *felis* RalF_CTD_ induced membrane ruffling and microvilli-like protrusions suggesting that the CTD plays a role in cytoskeletal rearrangements, similar to the known Arf6-GEF, EFA6 [71,72]. Furthermore, RalF_VPR_ (VPR-T4S), as well as the full-length VPR-containing ORF of *R*. *montanensis*, did not uniformly localize to the host plasma membrane, but instead were found strongly associated with intact actin stress fibers. Collectively, these results indicate that *R*. *typhi* and *R*. *felis* RalF proteins are similar to RalF_Rp_, with both the SCD and VPR required to spatially regulate Arf-GEF activities at plasma membrane/actin cytoskeletal junctions.

Remarkably, RalF_FL_ and RalF_CTD_ of *R*. *bellii* did not target the plasma membrane, yet instead showed perinuclear localization reminiscent of ectopically expressed RalF_L_. As *R*. *bellii* RalF_VPR_ associated with intact actin stress fibers, these data collectively indicate that the SCD alone is sufficient to localize RalF_Rb_ to the host cytoplasm. Visualization of the SCD sequence alignment across all RalF_L_ and RalF_R_ proteins revealed that RalF_Rb_ lacks three separate insertions within the SCD that are conserved in all other RalF_R_ proteins (S3B Fig). Thus, from a structural perspective, the SCD of RalF_Rb_ is more similar to RalF_L_ proteins than RalF_R_ proteins, which could explain why the SCD of *R*. *bellii* localizes to the perinuclear region of the cytoplasm. This is consistent with *R*. *bellii* sharing more genomic attributes with *Legionella* spp. [73], as well as being able to grow in various amoeba species unlike most other *Rickettsia* spp. (see Discussion).

**Fig 4:**
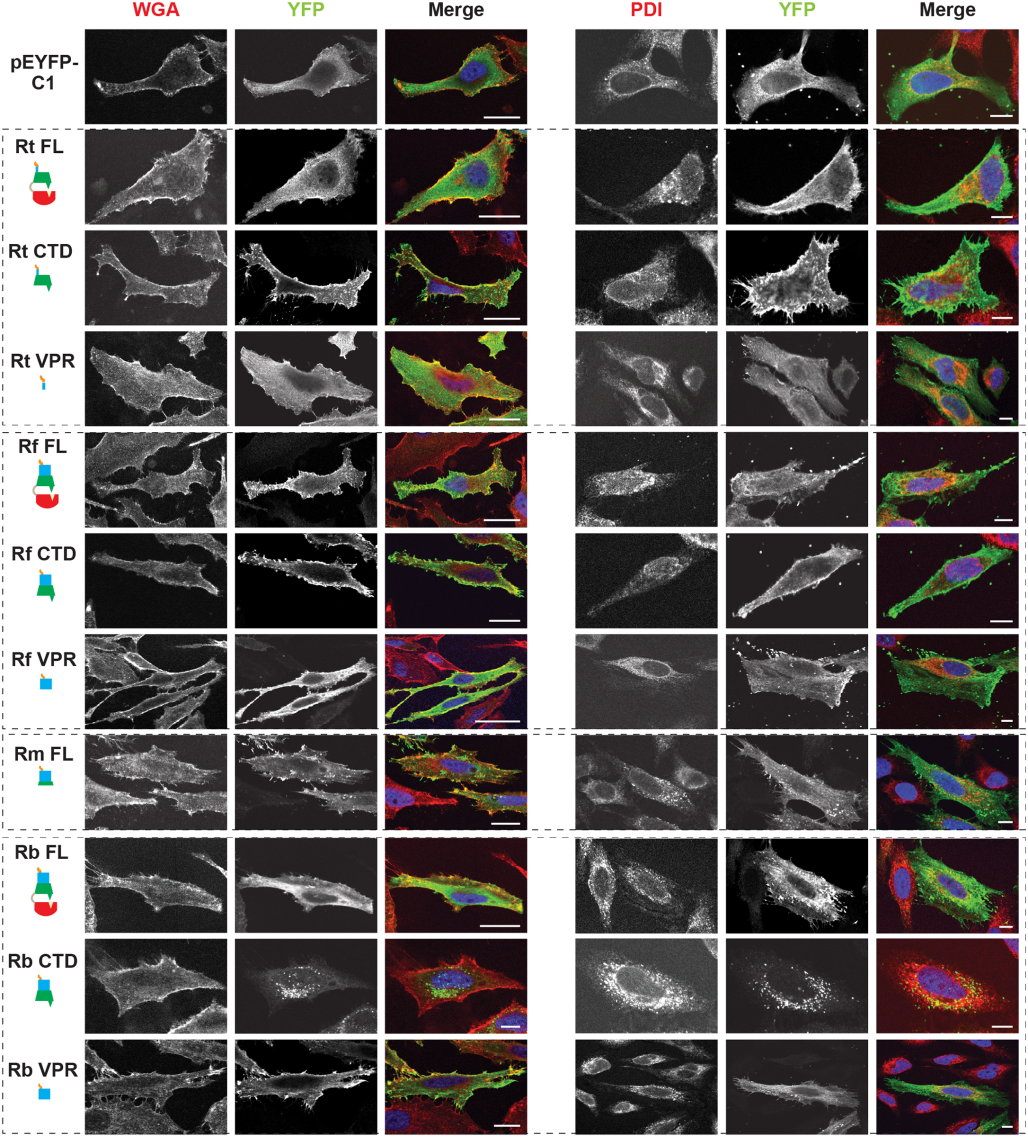
Subcellular localization of rickettsial RalF proteins to host membranes. HeLa cells expressing YFP tagged RalF proteins (green, described in Fig 2) were fixed and stained with Alexa Fluor 594 wheat germ agglutinin (WGA) to detect the plasma membrane (left) or anti-PDI antibody to detect the endoplasmic reticulum (right). DAPI (blue) is shown in the merged image. (Scale bar: 10 μm).

RalF membrane localization was further confirmed using two independent approaches. First, membrane fractionation of HeLa cells transfected with RalF-expressing plasmids revealed that all RalF_CTD_ proteins were predominately enriched in the membrane fraction, with RalF_FL_ and RalF_VPR_ proteins having less membrane enrichment (Fig 3 and S6 Fig). Second, RalF transfected cells were stained with Alexa Fluor 594 wheat germ agglutinin to detect plasma membrane or probed with anti-PDI (endoplasmic reticulum) or anti-GM130 (Golgi apparatus) antibodies (Fig 4 and S7 Fig), with the Pearson’s correlation coefficients calculated to measure co-localization with the respective membrane markers (S8 Fig). RalF_CTD_ of *R*. *typhi*, *R*. *felis*, *R*. *montanensis* indicate localization to the plasma membrane, while *R*. *bellii* RalF_CTD_ localized to the endoplasmic reticulum membrane recapitulating results observed with labeling host cell actin (Fig 3). Collectively, these data demonstrate the affinities of RalF_R_ proteins for host membranes, identifying the SCD as the major determinant for membrane localization, combined with the targeting of actin cytoskeleton by the VPR (Table 1).

**Table 1:** *Rickettsia* RalF domain characterization.

	Plasma Membrane Localization^a^	Perinuclear Localization^a^	Actin Binding^a^	Stress Fibers^b^	Membrane Ruffles^b^
Rt FL	+	-	+	+	-
Rt CTD	+	-	+	-	+
Rt VPR	-	-	+	+	-
Rf FL	+	-	+	+	-
Rf CTD	+	-	+	-	+
Rf VPR	-	-	+	+	-
Rm FL	-	-	+	+	-
Rb FL	-	+	+	+	-
Rb CTD	-	+	-	+	-
Rb VPR	-	-	+	+	-

Summary of protein localization, actin binding, stress fiber disruption and membrane ruffle formation for each RalF protein based on immunofluorescence and densitometry data (Figs 3, 4, and S6, S7 and S8 Figs).

^a^ +; association, -; negligible association

^b^ +; present, -; absent

Finally, for *R*. *typhi*, we monitored the subcellular localization of its RalF_CTD_ construct lacking the T4S (RalF_RtCTDΔT4S_). We observed indistinguishable localization patterns between RalF_RtCTD_ and RalF_RtCTDΔT4S_ (S4C Fig), suggesting that the T4S has no effect on localization or stress fiber disruption. In conjunction with results above (Fig 1A and 1B), these data bolster the role of the T4S of RalF_R_ proteins as an *rvh* T4SS translocation signal.

### Plasma membrane localization of RalF_Rt_ is dependent on PI(4,5)P_2_

**Fig 5:**
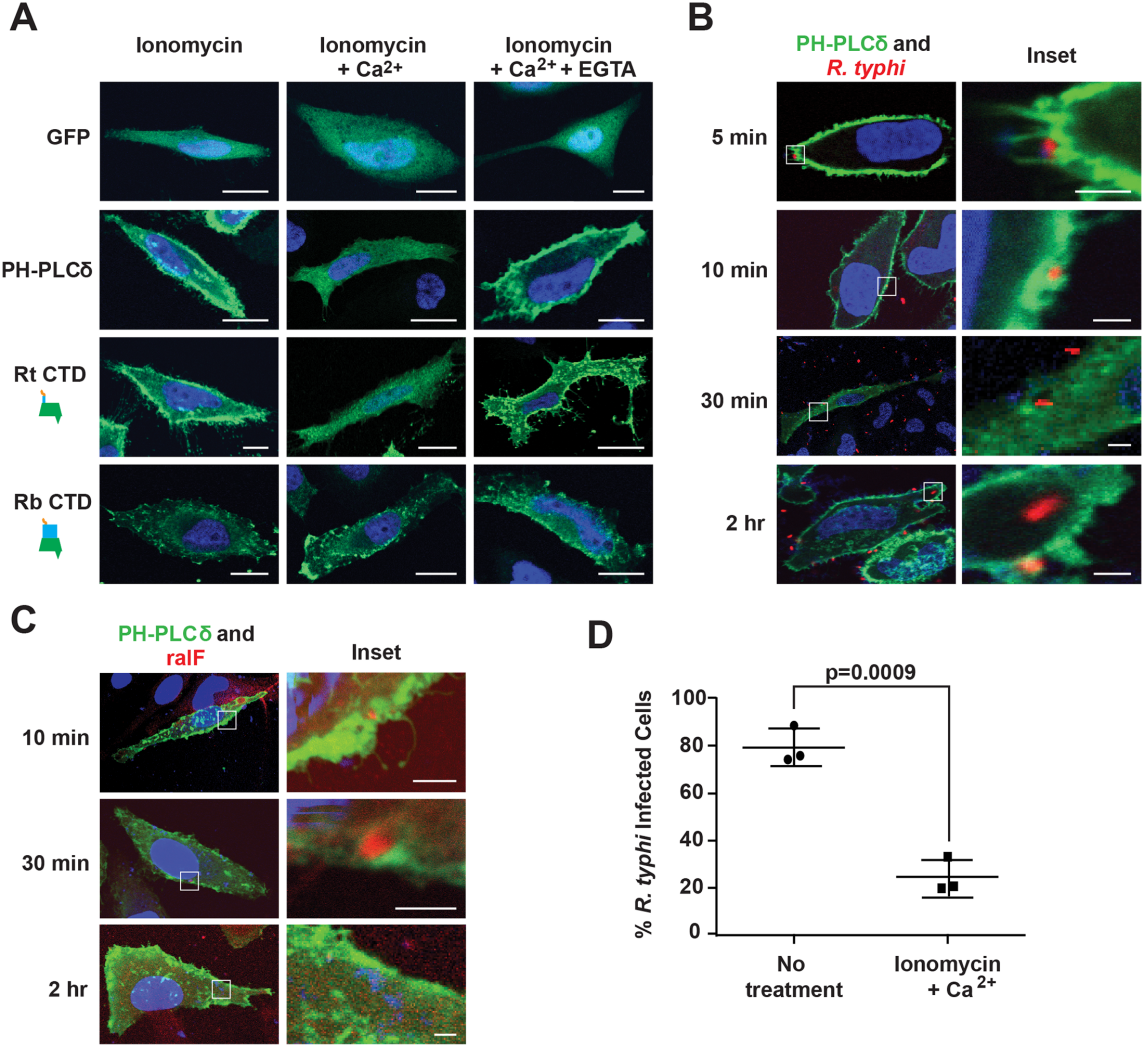
PI(4,5)P_2_ interacts with RalF_Rt_ and mediates *R*. *typhi* infection. (A) RalF_RtCTD_ co-localizes with PI(4,5)P_2_. HeLa cells transfected with pEYFP-C1 empty vector, GFP-C1-PLCδ-PH (a PI(4,5)P2 biosensor), EYFP–RalF_RtCTD_, or EYFP–RalF_RbCTD_ were treated with 5 μM ionomycin alone, with Ca^2+^, or with Ca^2+^ and EGTA. Nuclei were stained with DAPI (blue). (Scale bar: 10 μm). (B) PI(4,5)P_2_ is recruited during *R*. *typhi* infection. HeLa cells transfected with GFP-C1-PLCδ-PH (green) were infected with *R*. *typhi* (MOI ~100:1) for indicated times. *R*. *typhi* was detected with rat anti-*R*. *typhi* serum and Alexa Fluor 594 anti-rat antibody (red). Nuclei were stained with DAPI (blue). Boxed regions are enlarged to show detail (inset). (Scale bar: 1 μm). (C) RalF localizes to PI(4,5)P_2_-enriched regions of the plasma membrane during *R*. *typhi* infection. HeLa cells transfected with GFP-C1-PLCδ-PH (green) were infected with *R*. *typhi* (MOI ~100:1) for indicated times. RalF_Rt_ was detected with rabbit anti-RalF_Rt_ and Alexa Fluor 594 anti-rabbit antibodies (red). Nuclei were stained with DAPI (blue). Boxed regions are enlarged to show detail (inset). (Scale bar: 1 μm). (D) Ionomycin and Ca^2+^ treatment decreases *R*. *typhi* infection. HeLa cells treated with 5 μM ionomycin and Ca^2+^ or no treatment were infected with *R*. *typhi* (MOI ~100:1) for 2 hrs. *R*. *typhi* was detected with rat anti-*R*. *typhi* serum and Alexa Fluor 488 anti-rat antibody. Cell membrane was stained with Alexa Fluor 594 wheat germ agglutinin. The number of infected host cells was counted, with percent infection of three independent experiments (100 host cells counted for each) plotted. Error bars represent mean ± SD (Student’s two-sided t-test).

Previous studies showed the SCD of RalF_Rp_ has affinities for negatively-charged phospholipids; i.e., phosphatidylinositol 4,5-bisphosphate (PI(4,5)P_2_) and phosphatidylinositol 3,4,5-trisphosphate (PI(3,4,5)P_3_) [52,53]. Given the enrichment of PI(4,5)P_2_ at host membranes during early stages of phagocytosis [74], we evaluated the role of PI(4,5)P_2_ in RalF_R_ localization. As a baseline, we utilized the standard phospholipase C (PLC)-mediated catalyzation of PI(4,5)P_2_ within the IP3/DAG pathway of host cells [75]. Specifically, in the presence of ionomycin and Ca^2+^, PI(4,5)P_2_ is hydrolyzed to inositol 1,4,5-trisphosphate and diacylglycerol via PLC isozymes that regularly deplete the plasma membrane of PI(4,5)P_2_ following its role as a substrate in many signaling pathways [76]. To test PI(4,5)P_2_-dependent localization of RalF_Rt_ to the plasma membrane, HeLa cells ectopically expressing RalF_RtCTD_ were treated with ionomycin and Ca^2+^, with the distribution pattern of RalF_RtCTD_ monitored by immunofluorescence. With ionomycin and Ca^2+^ treatment, RalF_RtCTD_ becomes cytosolic compared to plasma membrane localization in the presence of ionomycin alone (Fig 5A). Upon treatment with EGTA, which chelates Ca^2+^, PI(4,5)P_2_ accumulates and RalF_RtCTD_ returns to the plasma membrane. HeLa cells expressing GFP-C1-PLCδ-PH, a biosensor of PI(4,5)P_2_, were used as a positive control to demonstrate the hydrolysis of PI(4,5)P_2_ in the presence of ionomycin and Ca^2+^. Additionally, RalF_RbCTD_ subcellular localization was shown to be unaffected by PI(4,5)P_2_ hydrolysis, consistent with its perinuclear distribution in host cells. Collectively, these data indicate that PI(4,5)P_2_ enrichment at the host plasma membrane is a requirement for efficient recruitment of RalF_Rt_, and probably also RalF_Rf_, given its similar subcellular localization pattern.

### PI(4,5)P_2_ recruitment is critical for *R*. *typhi* invasion of host cells

Phosphatidylinositols enriched at the host plasma membrane often play a critical role in bacterial internalization [74]. Given that RalF_Rt_ is expressed early (Fig 1F) and required (Fig 1G) for host invasion, and its localization to the host plasma membrane requires PI(4,5)P_2_ enrichment (Fig 5A), we sought to determine if PI(4,5)P_2_ is recruited by RalF_Rt_ during *R*. *typhi* infection. PI(4,5)P_2_ localization during *R*. *typhi* invasion was analyzed using immunofluorescence microscopy with GFP-C1-PLCδ-PH as a biosensor of PI(4,5)P_2_ localization. During early infection (i.e. 5 and 10 min post infection), PI(4,5)P_2_ was highly localized to pseudopodia at the *R*. *typhi* entry foci (Fig 5B). As internalization progressed, *R*. *typhi* was surrounded by a vacuole with diminished PI(4,5)P_2_ localization. Once *R*. *typhi* detached from the membrane, it was no longer associated with PI(4,5)P_2_. Furthermore, detection of RalF_Rt_ during the infection process revealed co-localization of PI(4,5)P_2_ and RalF_Rt_ during early infection (Fig 5C). In agreement with RalF_Rt_ early expression, which diminished at later stages of infection (Fig 1F), PI(4,5)P_2_ recruitment decreased as infection progressed.

Finally, we evaluated whether or not the recruitment of PI(4,5)P_2_ to *R*. *typhi* entry foci is critical for *R*. *typhi* infection. Pretreatment of HeLa cells with ionomycin and Ca^2+^ to deplete PI(4,5)P_2_ from the membrane prior to infection resulted in a significant decrease in *R*. *typhi* infection (Fig 5D), strengthening the evidence that PI(4,5)P_2_ is a target molecule involved in RalF_Rt_-associated host cell invasion.

### RalF_Rt_ interacts with Arf6 but not Arf5

**Fig 6:**
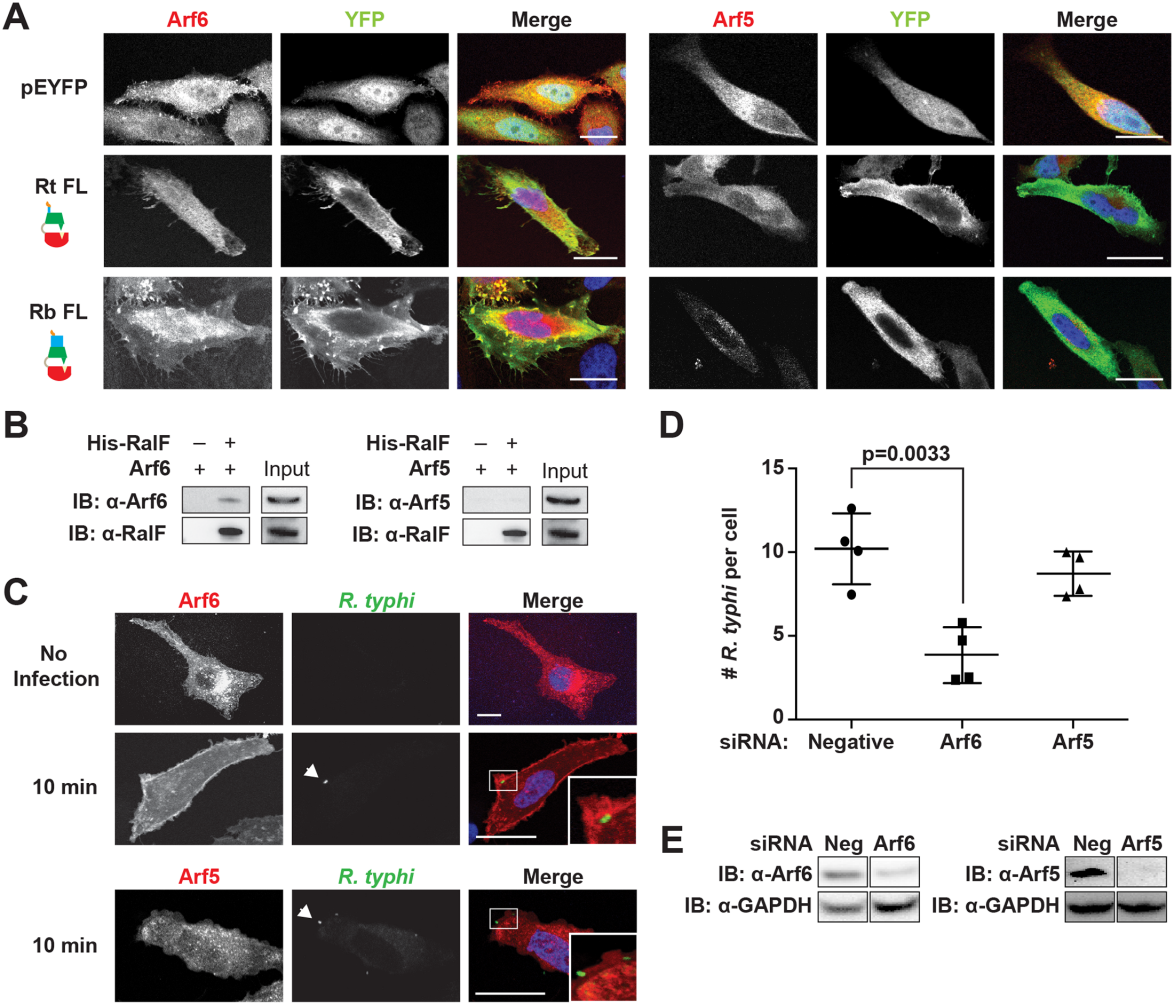
Arf6 is recruited by *R*. *typhi* RalF and is required for infection. (A) Ectopically expressed RalF_RtFL_ co-localizes with Arf6 but not Arf5. HeLa cells co-expressing EYFP, EYFP-RalF_RtFL_ or EYFP-RalF_RbFL_ and mRFP-Arf6 (left) or -Arf5 (right) were fixed with 4% para-formaldehyde. Nuclei were stained with DAPI (blue). (Scale bar: 10 μm). (B) RalF_RtFL_ pull-down of Arf6. Lysates from HEK293T cells expressing mRFP-Arf5 or -Arf6 were incubated with HisPur Cobalt resin bound with rHis-RalF_RtFL_ or resin alone. Bound proteins were eluted with imidazole and analyzed by protein immunoblot using antibodies as indicated. (C) Arf6 is recruited during *R*. *typhi* entry. HeLa cells expressing mRFP-Arf5 or -Arf6 (red) were infected with partially purified *R*. *typhi* (MOI ~100). Ten minutes post infection, cells were fixed and *R*. *typhi* detected with anti-*R*. *typhi* serum (green). DAPI (blue) is shown in the merged image. Boxed regions are enlarged to show detail. White arrowheads indicate *R*. *typhi*. (Scale bar: 5 μm). (D) Arf6 knockdown inhibits *R*. *typhi* infection. HeLa cells transfected with negative, Arf6, or Arf5 siRNA were infected with partially purified *R*. *typhi* (MOI ~100). At 2 hrs post infection, cells were fixed, plasma membrane stained with Alexa Fluor 594 wheat germ agglutinin, and *R*. *typhi* detected with rat anti-*R*. *typhi* serum and Alexa Fluor 488 anti-rat antibody. The number of *R*. *typhi* per host cell was counted for 100 host cells for three independent experiments. Error bars represent mean ± SD (Student’s two-sided t-test). (Scale bar: 5μm). (E) Confirmation of Arf6 and Arf5 knockdown. Arf6 and Arf5 knockdown, 80% and 96% respectively, was confirmed by western blot and densitometry analysis using ImageJ (NIH).

The Arf-GEF activity of RalF_L_ is activated upon membrane binding, with Arf1 the preferred substrate [49,52]. Arf1 is predominantly localized to the Golgi apparatus and plays a role in intra-Golgi transport [77]. Given the association of RalF_Rt_ with the plasma membrane, we hypothesized that it might instead recruit Arf6, which is predominantly localized to the plasma membrane where it is involved in endocytosis, endosomal recycling and exocytosis of secretory granules [78–81]. Using immunofluorescence, RalF_RtFL_ was found to recruit Arf6 but neither Arf5 (Fig 6A) nor Arf1 (S9 Fig) to the plasma membrane. Arf5 localizes primarily to the endoplasmic reticulum/Golgi intermediate compartment and the cis-Golgi, where it regulates endoplasmic reticulum to Golgi transport; therefore, Arf5 was used as a negative control [82]. Interestingly, RalF_RbFL_ was similarly found to co-localize with Arf6 but not Arf5 or Arf1 in the perinuclear space.

To further confirm a RalF_RtFL_ and Arf6 interaction, a protein pull-down assay was performed. Using rHis-RalF_RtFL_ as bait and mRFP-Arf5 or mRFP-Arf6 as the prey, we confirmed that RalF_RtFL_ interacted with Arf6 and not Arf5 (Fig 6B).

### Arf6 recruitment is critical for *R*. *typhi* invasion of host cells

Activation of Arf6 at the plasma membrane drives the recruitment of phospholipase D and phosphatidylinositol 4-phosphate 5-kinase (PIP5K), which ultimately results in actin remodeling [83,84]. To determine if Arf6 is recruited during *R*. *typhi* entry, we used immunofluorescence microscopy to monitor Arf6 localization. As early as 10 min post infection, Arf6 was recruited to the plasma membrane at *R*. *typhi* entry foci, while Arf5 remained cytoplasmic (Fig 6C). Given that RalF_Rt_ localizes with Arf6 at plasma membrane (Fig 6A) and recruits Arf6 at the *R*. *typhi* entry foci (Fig 6C), we predicted that knockdown of Arf6 would decrease *R*. *typhi* infection. Indeed, siRNA-mediated Arf6 knockdown significantly decreased the number of *R*. *typhi* per cell, while Arf5 knockdown had no significant effect on *R*. *typhi* infection(Fig 6D and 6E). These results indicate that RalF_Rt_ recruits Arf6 at the plasma membrane during early infection, with spatially regulated Arf-GEF activity required for host cell invasion.

## Discussion

Bacteria invading eukaryotic cells employ diverse strategies to subvert the host cellular actin cytoskeleton, allowing for internalization into normally non-phagocytic host cells [85]. For some bacterial species, surface proteins bind host cell receptors and trigger an “outside-in” signaling cascade, which induces cytoskeletal rearrangements and recruits the endocytic machinery to entry foci [86]. Such receptor-mediated induction of bacterial uptake is a strategy employed by *Listeria monocytogenes*, which utilizes two adhesins (InlA and InlB) to bind host proteins (E-cadherin, receptor gC1qR, proteoglycans) and activate the tyrosine kinase receptor Met [87]. Invasive species of *Yersinia* also employ two adhesins (invasin and YadA) to bind a subset of β1-integrin host receptors, facilitating invasion that is dependent on signaling from the Rho GTPase Rac1 and activation of the actin nucleating complex Arp2/3 [88,89]. Alternatively, other bacterial species translocate effectors into host cells to initiate actin remodeling and facilitate bacterial uptake. For example, *Salmonella typhimurium* utilizes its type III secretion system to inject host cells with the effector SopE, which stimulates GDP/GTP nucleotide exchange on Rho GTPases Rac1 and Cdc42, resulting in membrane ruffling and actin cytoskeleton rearrangement [90]. While a receptor-mediated process has been previously characterized for *R*. *conorii* invasion of mammalian cells (discussed below), to date no secreted effectors for any *Rickettsia* species have been characterized for their role in inducing uptake into host cells.

While most species of the order Rickettsiales encode the *rvh* T4SS [91], effectors have only been identified for some species of the family Anaplasmataceae. For *Anaplasma phagocytophilum*, *rvh* effectors are translocated to the mitochondria (Ats-1) and nucleus (AnkA) to inhibit etoposide-induced apoptosis and down-regulate host defense genes, respectively [92–94]. AM185, AM470, AM705 (AnkA), and AM1141 have been identified as putative *rvh* T4SS effectors of *Anaplasma marginale* using a heterologous T4SS (*L*. *pneumophila dot*/*icm*), yet none have been characterized for their roles in invasion [95]. *Ehrlichia chaffeensis* utilizes the *rvh* T4SS to translocate the effector ECH0825 into host mitochondria, resulting in inhibition of Bax-induced apoptosis [96]. Herein, we identified RalF as the first *rvh* T4SS effector for species in the family Rickettsiaceae. We provide evidence that *R*. *typhi* RalF interacts with RvhD4, the *rvh* T4SS coupling protein that presumably recognizes effectors and regulates their translocation similar to VirD4 proteins of other P-type T4SSs. Treatment of purified *R*. *typhi* with proteinase K degraded surface exposed RalF, providing further evidence that RalF is secreted. Furthermore, using immunofluorescence we show that RalF_Rt_ is expressed early during host cell invasion. Because RalF is expressed early during invasion, we hypothesized that RalF is critical for invasion, which was confirmed using antibody pretreatment assays.

Prior studies comparing the subcellular localization of ectopic RalF_L_ and RalF_Rp_ determined that, despite strong conservation in the S7D and SCD across these proteins, cryptic signatures within the SCD targeted these proteins to different host membranes [52,53]. RalF_Rp_ localization to the plasma membrane, mediated by elevated positively charged residues within the lipid sensor of the SCD, was anticipated to be true for other RalF_R_ proteins, given the strong sequence conservation within the SCD across RalF_R_ homologs. Furthermore, despite extensive variation within the VPR across RalF_R_ proteins, the presence of proline-rich regions in all proteins suggested that this region likely encodes a conserved motif that facilitates interaction with the host cytoskeleton, as was shown for RalF_Rp_ [52,53]. Indeed, our co-localization assays confirmed that, for RalF of *R*. *typhi* and *R*. *felis*, the SCD mediates interaction with the host plasma membrane, with the VPR facilitating interaction with the host cytoskeleton.

In contrast, the perinuclear localization of RalF of *R*. *bellii*, reminiscent of the localization of ectopic expressed RalF_L_ proteins at the host secretory network, was unexpected. The VPR of RalF_Rb_ is similar in length to VPRs of RalF proteins from *R*. *felis*, *R*. *akari* and *R*. *australis*, with all of these proteins predicted to encode a coiled-coil motif typical of some eukaryotic Arf-GEFs; e.g., EFA6 [71]. While containing an extraordinary number of Pro residues, the VPR of RalF_Rb_ nonetheless targets the host cytoskeleton, suggesting that other characteristics of the protein mediate its localization to the cytoplasm. Indeed, *in silico* analyses revealed three conserved insertions within the SCD of RalF_R_ proteins that are absent from RalF_Rb_. Furthermore, relative to all RalF proteins, the S7D of RalF_Rb_ contains an odd insertion as well as a slightly less hydrophobic active site (S2 Fig), the significance of which is unknown. It is tempting to speculate that the perinuclear localization of RalF_Rb_ reflects a unique cytosolic lifestyle of *R*. *bellii*, an ancestral lineage with a different genomic repertoire relative to other *Rickettsia* species [51] and the unique ability to grow and survive in several species of amoeba [73]. As *R*. *bellii* has been observed invading nuclei of mammalian cells *in vitro* [73], RalF may play a role in this process, though other *Rickettsia* species that lack RalF also are known to invade host cell nuclei [97]. Notwithstanding, the SCD-driven perinuclear localization of RalF_Rb_ might thus be considered the retention of an ancestral role for RalF_R_ proteins in targeting host vesicular trafficking, similar to RalF_L_ proteins. Collectively, our detailed dissection of the domain requirements for subcellular localization strongly implies differential utilization of Arf-GEF activities for those species of *Rickettsia* that encode RalF.

RalF_Rb_ aside, the subcellular localization of other RalF_R_ proteins to the plasma membrane suggested Arf6 might be their preferred host target, given the predominant localization of Arf6 to the plasma membrane [98] and a previous study showing that RalF_Rp_ can catalyze nucleotide exchange on Arf6 [52]. Arf6 activation by some intracellular pathogens (e.g., species of *Salmonella*, *Yersinia* and *Chlamydia*) is known to induce actin remodeling and mediate bacterial invasion via unique pathways. *Salmonella enterica* activates Arf6 to recruit the Arf-GEF ARNO, which in turn activates Arf1 to enable WASP family veroprolin homolog (WAVE) regulatory complex-dependent actin assembly [99]. Arf6 activation by species of *Yersinia* and *Chlamydia* leads to activated PIP5K, which converts PI(4)P to PI(4,5)P_2_ at the plasma membrane [100,101]. As PI(4,5)P_2_ enrichment at the host plasma membrane modulates many actin-binding proteins, including α-actinin, talin, vinculin, gelsolin, and the WASP-Arp2/3 complex [102–107], effector-driven accumulation of this phosphatidylinositide can be considered a strategy for induction of phagocytosis.

**Fig 7:**
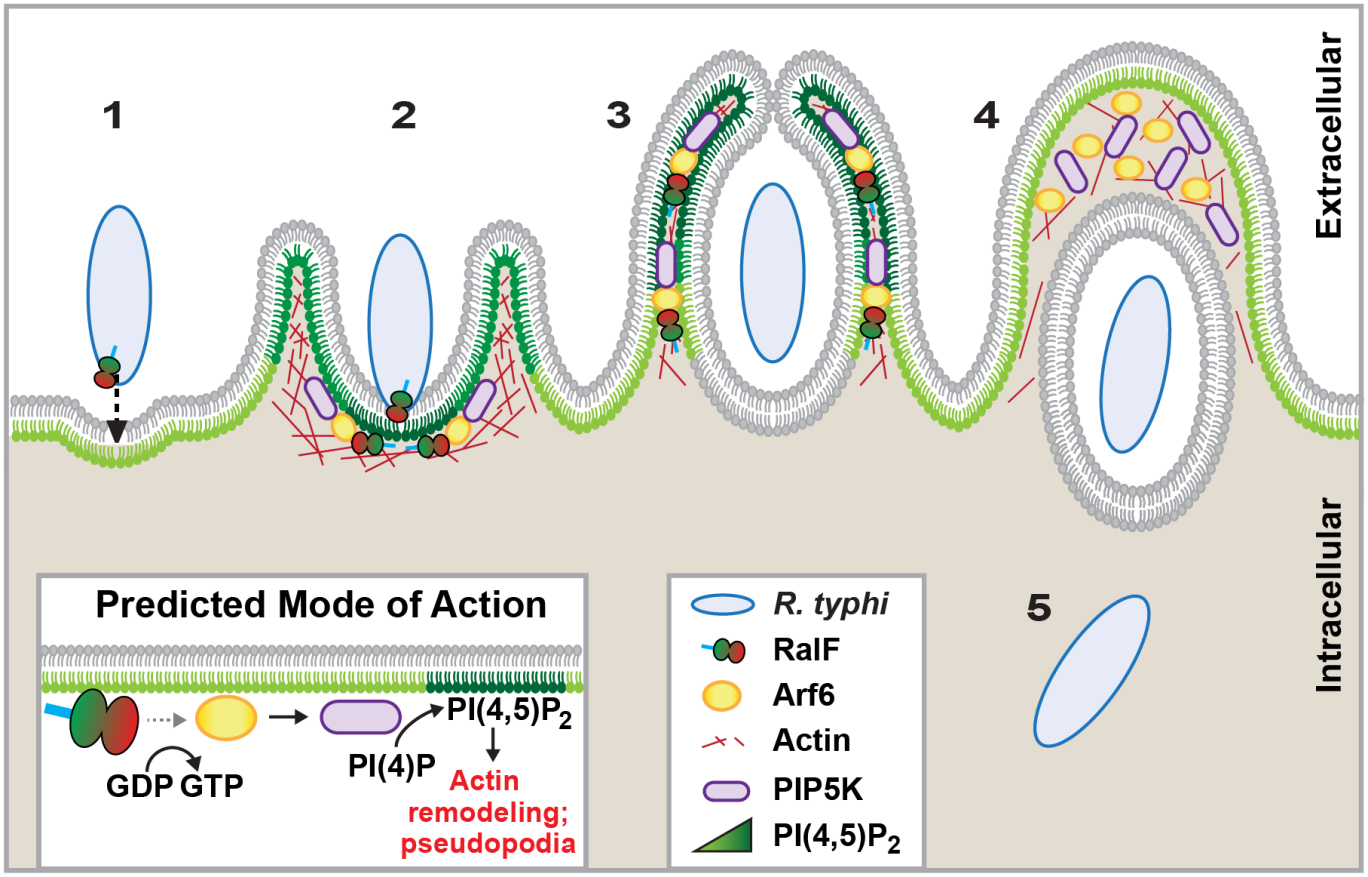
Schematic of *R*. *typhi* entry. *R*. *typhi* entry has been broken down into five conceptual stages: binding (1); extension of pseudopodia (2); membrane fusion and internalization (3); formation of early endosome (4); bacterial escape from endosome (5). Schematic is a representation of micrographs from Figs 5B, 5C and 6C. Inset depicts hypothetical recruitment and activation of PIP5K via RalF_Rt_-activated Arf6, which results in PI(4,5)P_2_ enrichment and actin rearrangement to facilitate for *R*. *typhi* entry.

Given that *R*. *typhi* secretes RalF early during host cell invasion, we hypothesized that this Arf-GEF recruits Arf6 to entry foci, precipitating the enrichment of PI(4,5)P_2_ at the plasma membrane to facilitate bacterial invasion. Indeed, our *in vitro* and *in vivo* results confirm that Arf6 co-localizes with RalF and *R*. *typhi* at entry foci. Additionally, during *R*. *typhi* infection, PI(4,5)P_2_ noticeably accumulated in the membranes of pseudopodia, with a decreased concentration at the base of the phagocytic cup as internalization progressed. Furthermore, the role of PI(4,5)P_2_ in bacterial internalization was bolstered by the significant reduction in *R*. *typhi* invasion upon PI(4,5)P_2_ hydrolysis. Thus, an increase in PI(4,5)P_2_ induced by rickettsial RalF activation of Arf6 is predicted to initiate actin remodeling and ultimately facilitate bacterial invasion (Fig 7). Remarkably, this function for RalF_R_ is markedly different than RalF_L_, which is utilized by *L*. *pneumophila* to recruit Arf1 to the LCV [49]. Unlike species of *Legionella*, *Rickettsia* species do not reside in vacuoles but rather lyse the phagosome and replicate within the host cytoplasm. Thus, despite carrying a similar Arf-GEF that is unknown from any other bacteria, different intracellular lifestyles across species of *Rickettsia* and *Legionella* have driven divergent roles for RalF during bacterial infection.

**Fig 8:**
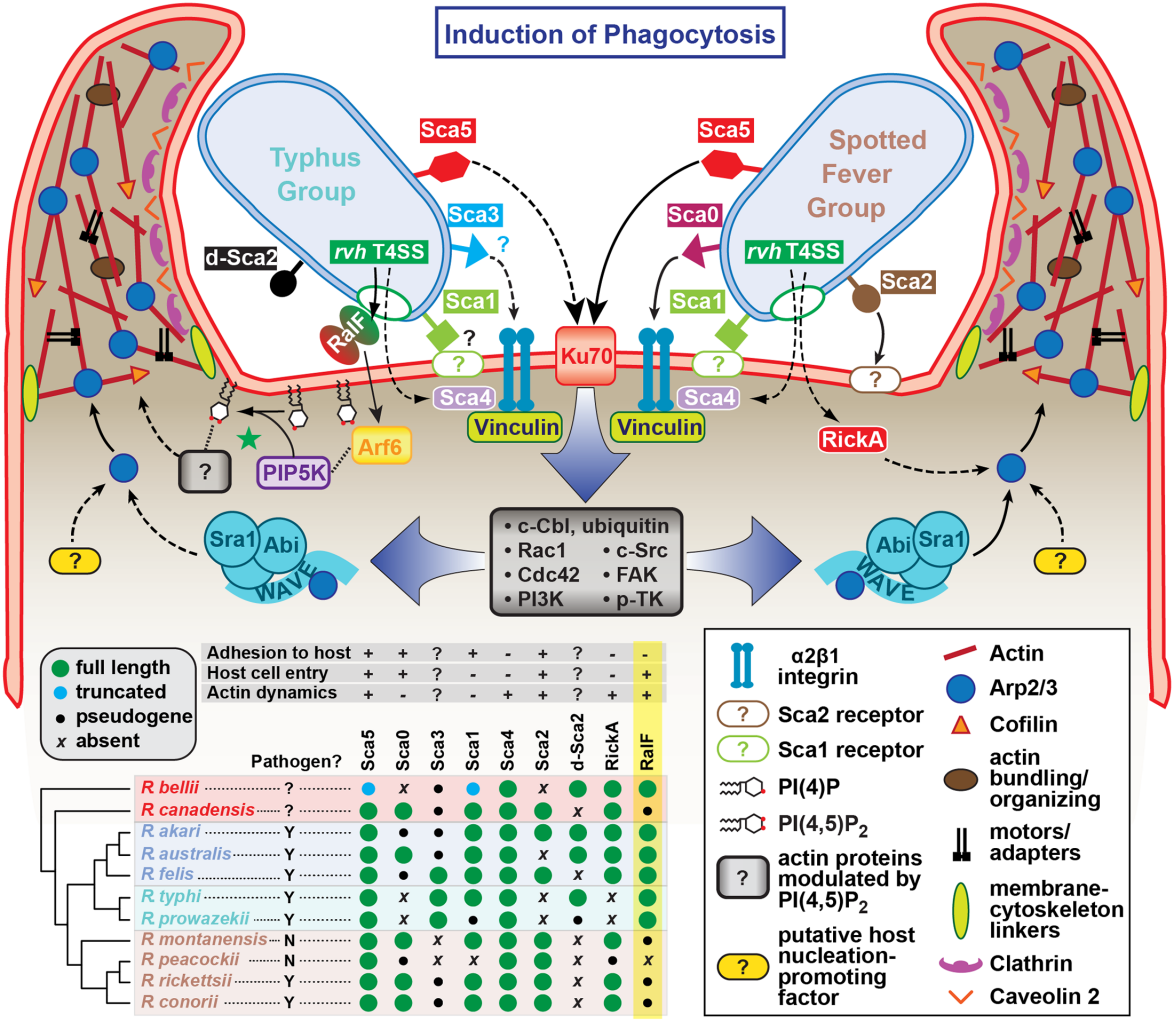
Model for the variable pathways utilized by divergent *Rickettsia* species for host cell entry. General pathways for Typhus Group (TG, left) and Spotted Fever Group (SFG, right) rickettsiae species are inferred primarily from previous work on SFG rickettsiae species *R*. *conorii* [26] and *R*. *parkeri* [108] or data from the present study (*R*. *typhi*). At center, a conserved proximal hub of the pathway commences with Sca5 binding to host receptor Ku70 [110], which triggers a host-signaling cascade (gray box) involving c-Cbl-mediated ubiquitination of Ku70, Rho-family GTPases Cdc42 and Rac1, phosphoinositide 3-kinase (PI3K) activity, and activation of tyrosine kinases (e.g., c-Src, FAK and p-TK) and their phosphorylated targets. The divergent distal arms of this pathway involve recruitment of factors for activating the actin nucleating complex (Arp2/3), which leads to host actin polymerization, extensive membrane ruffling and filopodia formation, and bacterial internalization in a clathrin and calveolin dependent process. For SFG rickettsiae, the WAVE complex recruits Arp2/3, with its activation via an unknown nucleation promoting factor (either host or bacterial; e.g., RickA). While these processes remain to be characterized for TG rickettsiae, our work suggests that secreted RalF recruits the GTPase Arf6, precipitating an accumulation of PI(4,5)P_2_ that modulates the activities of a range of actin-associated host proteins (green star). Additional bacterial proteins, some of which are known to facilitate host cell entry, have white lettering with colored boxed backgrounds. Known pathways for protein secretion and host cell receptor-binding, as recently reviewed [45], are shown with solid black lines; all other modeled pathways (shown with dashed lines) are either inferred by homology (e.g., Sca1 of TG rickettsiae as an adhesin based on characterization for Sca1 of *R*. *conorii* [36]) or estimated based on *in silico* analyses (e.g., Sca3 of TG rickettsiae as a putative analog to the α2β1 integrin-binding Sca0 of *R*. *conorii* [35]). A phylogenomics analysis across select *Rickettsia* species (bottom, left) illustrates the genomic variation underlying all of the bacterial components of the models. Adapted from our recent report on the *Rickettsia* secretome [45]. Red, ancestral group (AG); blue, transitional group (TRG); aquamarine, TG; brown, SFG.

Currently, the predominant knowledge of rickettsia entry and invasion of host cells is based on studies of species from SFG rickettsiae, whereby the surface antigen Sca5 binds host receptor Ku70 to activate a signaling cascade leading to Arp2/3 activation and ultimately actin polymerization, membrane rearrangement and bacterial invasion [26,108,109]. The conservation of Sca5 across all *Rickettsia* species implies that this receptor-mediated mechanism for entry is likely conserved (Fig 8) [45]. However, depletion of host Rho family GTPases and nucleation-promoting factors that activate Arp2/3 has only a modest effect on rickettsial invasion, suggesting there are other bacterial or host proteins that activate Arp2/3 during infection [108]. Most species of SFG rickettsiae encode an Arp2/3 activating protein, RickA, which could potentially play this role, although its secretion during infection has yet to be demonstrated. Interestingly, genes encoding RickA are absent from species of TG rickettsiae (*R*. *typhi* and *R*. *prowazekii*); thus, if bacterial Arp2/3 activators are a requirement for invasion, factors other than RickA must be utilized for species of TG rickettsiae. Accordingly, we propose that RalF plays a role in host actin rearrangement and bacterial invasion.

Aside from the Sca5-Ku70 interaction and subsequent downstream signaling cascade, other rickettsial adhesins have been characterized for facilitating host cell invasion (Fig 8). However, the lack of conservation of these adhesins (e.g., Sca0 and Sca2) across diverse *Rickettsia* species implies the existence of multiple mechanisms for rickettsial host cell invasion [45]. It is also probable that each species likely encodes redundancy for factors that facilitate entry, and that some factors may selectively operate for invasion of specific cells (arthropod versus mammalian) throughout the complex rickettsial lifecycle. Thus, it is probable that lineage-specific factors are employed by different species of *Rickettsia* to successfully invade and colonize diverse eukaryotic cells. Our identification of lineage-specific Arf-GEF utilization across diverse rickettsial species exemplifies this, and illuminates previously unappreciated mechanisms for host cell invasion and infection.

## Materials and Methods

### Bacterial strains, cell culture, and infection

Vero76 (African green monkey kidney, ATCC: CRL-1587), HEK293T and HeLa (ATCC: CCL-2) cells were maintained in minimal Dulbecco’s Modified Eagle’s Medium (DMEM with 4.5 gram/liter glucose and 480 L-glutamine; Mediatech, Inc.) supplemented with 10% heat inactivated fetal bovine serum (FBS) at 37°C with 5% CO_2_. *R*. *typhi* strain Wilmington (ATCC: VR-144) was propagated in Vero76 cells grown in DMEM supplemented with 5% heat inactivated fetal bovine serum at 34°C with 5% CO_2_. Rickettsiae were partially purified as previously described [111]. Infections with *R*. *typhi* were performed 18–24 hrs post transfection with a multiplicity of infection (MOI) of ~100:1. For antibody pretreatment experiments, partially purified *R*. *typhi* was incubated with 20 μg Melon Gel IgG (Thermo Scientific) purified rabbit pre-immune serum or anti-RalF_Rt_ polyclonal antibody or 20 μg purified rabbit pre-immune serum or anti-RalF_Rt_ Fab fragments for 30 min prior to infection. Fab fragments were purified using the Fab Purification Kit (Thermo Scientific) according to manufacture’s protocol.

### Recombinant protein purification and antibody production

The expression and purification of recombinant proteins were performed as previously described [111]. Codon optimized (Life Technologies) *R*. *typhi rvhD4* (RT0284) was cloned into pTrcHis2-TOPO vector under the control of the *trc* promoter (Life Technologies). Full-length *R*. *typhi ralF* was cloned into pEXP5-NT/TOPO (Life Technologies) and transformed into *E*. *coli* strain bl21-codonplus(de3)-ril (Stratagene). Primers used for cloning can be found in S1 Table. The expression of recombinant proteins was induced with 1 mM IPTG and recombinant proteins were purified by affinity chromatography under native conditions using nickel-nitrilotriacetic acid resin (Ni-NTA) superflow columns (Qiagen) according to manufacturer’s instructions. Polyclonal antibody was generated in rabbit using recombinant RalF_RtFL_ (Alpha Diagnostic Intl. Inc).

### Bacterial two-hybrid assay

*R*. *typhi* gene sequences (RT0362, GenBank accession no. YP_067323) encoding full-length RalF (RalF_RtFL_) and the *rvh* T4SS signal truncation (RalF_RtΔT4S_) were cloned into the pTRG “prey” plasmid (BacterioMatch II two-hybrid system; Stratagene). A codon optimized (Life Technologies) *R*. *typhi rvhD4* gene (RT0284, YP_067246) was cloned into the pBT “bait” plasmid. Primers used for cloning can be found in S1 Table. The bait (pBT-RvhD4) and prey (pTRG-RalF_RtFL_ or pTRG-RalF_RtΔT4S_) plasmids (100ng each) were co-transformed into BacterioMatch II reporter electrocompetent cells according to the manufacturer’s instruction (GenePulser Xcell, BioRad). The percent growth of CFUs of reporter cells harboring recombinant plasmids on dual selective screening medium were calculated relative to CFUs obtained on non-selective His dropout medium by a drop plate method for counting CFUs [112].

### VirD4 ATPase assay

RvhD4 ATPase activity was monitored using a Quantichrom ATPase/GTPase assay kit (Bioassay Systems), according to the manufacturer’s instructions and as described previously [113]. Briefly, 200–12.5 ng/well of purified recombinant RvhD4 protein was incubated in the presence of 1 mM ATP for 30 min at 37°C. Generated free phosphate was quantified by measuring absorbance at OD 620 nm. All of the samples were measured in triplicate wells, and data are given as averages ± S.D. of three independent experiments.

### Protease treatment of *R*. *typhi*

*R*. *typhi* was purified from heavily infected Vero76 cells. Briefly, infected cells were scrapped into media and spun at 12,000 x g for 10 min at 4°C. Cells were resuspended in ice cold PBS, pH 7.2 containing MgCl_2_ (PBS-Mg) and sonicated for 10 sec on ice using output 6 of a Sonic Dismembrator (Fisher Scientific). The lysate was filtered through a 5.0 μm filter (Millipore). The filtrate containing *R*. *typhi* was layered onto a 20% sucrose cushion at a 1:1 ratio and centrifuged at 16,000 x g for 15 min at 4°C to pellet *R*. *typhi*. *R*. *typhi* was resuspended in PBS-Mg and again purified with a 20% sucrose cushion. Purified *R*. *typhi* was treated with 400 μg/mL or 800 μg/mL Proteinase K (Sigma-Aldrich) for 1 hr at room temperature in PBS-Mg buffer as previously described [114]. Following incubation, Halt Protease and Phosphatase Inhibitor Cocktail (Thermo Scientific) was added to the reaction, and bacteria centrifuged at 16,000 x g for 10 min at 4°C. *R*. *typhi* were washed with PBS-Mg and resuspended in PBS-Mg and NuPAGE LDS sample buffer and reducing reagent (Life Technologies). Lysates were separated on a NuPAGE Bis-Tris SDS-gel (Life Technologies) and immunoblotted with rabbit anti-RalF or anti-EF-Ts as the *R*. *typhi* cytoplasmic marker [43,115]. Densitometry was performed using ImageJ (NIH) and RalF intensity was normalized to EF-Ts.

### Bioinformatics and phylogenomics analyses

Using RalF_Rt_ as a query, BLASTP searches were performed against the NCBI ‘*Rickettsia’* database (taxid:780). Full length RalF_R_ homologs were aligned with MUSCLE v3.6 [116] using default parameters. Initial domain characterization of RalF_R_ proteins followed that previously described for *R*. *prowazekii* [52]. Using Phyre v.2.0 [69], RalF_Rt_ was modeled to the crystal structures of *Legionella pneumophila* RalF (PDB 1XSZ, 4C7P) [50,53] to confirm the boundaries of the Sec7 domain (S7D) and Sec7-capping domain (SCD). The S7D and SCD of *Rickettsia* and *Legionella* RalF homologs were aligned with MUSCLE, superimposing the secondary structure of RalF_L_ over the alignment.

The divergent C-terminal domain (CTD) of RalF_R_ proteins was further described based on distinct characteristics, i.e. a sequence of variable length that includes a Pro-rich tract, as well as a putative secretion signal sequence within the terminal 40 aa. Additional *Rickettsia* proteins that lack the S7D and SCD, mostly from SFG rickettsiae, were utilized to characterize this region. All full length and partial RalF_R_ homologs were used to assess the synteny of the *ralF* locus across select *Rickettsia* genomes. For these genomes, gene neighborhood models were constructed using the Kyoto Encyclopedia of Genes and Genomes (KEGG) database [117], with manual adjustment to gene annotations. Additional bioinformatics/phylogenomics methodologies are described in S2–S5 Figs.

### Mammalian expression plasmids and transfections

Genomic DNA from *R*. *bellii* str. OSU 85–1299, *R*. *felis* str. Pedreira, *R*. *montanensis* str. M5/6, and *R*. *typhi* str. Wilmington was purified using DNeasy Blood and Tissue Kit (Qiagen). RalF constructs were amplified as EcoRI/BamHI fragments using primers in S1 Table, with the exception of RalF_RbFL_, which was cloned using Clontech InFusion technology. Amplicons were cloned into the pGEMT-Easy vector (Promega) and confirmed by sequencing (The Biopolymer/Genomics Core Facility, University of Maryland School of Medicine). Plasmids were digested with EcoRI and BamHI, with *ralF* fragments subcloned into the pEYFP-C1 vector (Clontech). All plasmids were transformed into Mix & Go Competent Cells—Strain Zymo 5α (Zymo Research). Plasmids pCDNA3-mRFP-Arf1, pCDNA3-mRFP-Arf5 and pCDNA3-mRFP-Arf6 were generous gifts from Prof. Vassilis Koronakis (University of Cambridge, UK). GFP-C1-PLCδ-PH (Addgene plasmid # 21179) was kindly gifted by Tobias Meyer [118]. All plasmids were purified using PerfectPrep EndoFree Plasmid Maxi Kit (5 Prime).

For transfections, HeLa cells seeded in 8-well chamber slides were transfected with 200ng plasmid per well using Xfect (Clontech) and HEK293T cells in T-75 flasks were transfected with 10μg plasmid using Lipofectamine 2000 (Life Technologies) according to manufactures’ protocols.

### Immunofluorescence

Twenty-four hours post transfection or at indicated times post infection, cells were fixed with 4% PFA for 10 min at room temperature. Cells were washed three times with PBS and permeabilized in Blocking Buffer (0.2% saponin, 5% FBS in PBS) for 30 min. Primary antibodies mouse anti-PDI (clone RL90, BD Transduction Laboratories, diluted 1:200), mouse anti-GM130 (clone 610822, BD Transduction Laboratories, diluted 1:200), rat anti–*R*. *typhi* serum (1:500), rabbit anti-RalF_Rt_ (1:100), and rabbit anti-GFP (Life Technologies, diluted 1:1000) were diluted in blocking buffer and incubated with cells for 1 h. Cells were then washed with PBS and incubated with Alexa Fluor 594 or Alexa Fluor 488 secondary antibodies (Life Technologies) diluted 1:2000 in Blocking Buffer for 1 h or 30 min. Finally, cells were washed three times with PBS and mounted using ProLong Gold Anti-Fade mounting media with DAPI (Life Technologies). Actin was stained with Alexa Fluor 594 phalloidin (Life Technologies) and the plasma membrane stained with Alexa Fluor 594 wheat germ agglutinin (WGA, Life Technologies) according to manufacturer’s protocol. For confocal microscopy, cells were viewed under a Zeiss LSM 510 Meta Confocal Microscope (University of Maryland Baltimore Confocal Core Facility). For conventional fluorescence microscopy a Nikon Eclipse E600 fluorescent microscope with a Q Imaging Retiga 2000R camera was used to capture images with QCapture Pro software. Images were processed using ImageJ software (NIH). Co-localization analysis was performed using the CoLoc2 plugin in the ImageJ software program [119]. The Pearson’s correlation coefficient was calculated for 5–10 cells per condition from two independent experiments to measure the strength of association between each RalF protein and the cell organelle (i.e., plasma membrane, endoplasmic reticulum, or Golgi apparatus). Two-sided Student’s t-tests were performed to determine statistical significance for co-localization coefficients compared to control eYFP.

### Cell fractionation

Cellular fractionation was completed as previously described [52]. Briefly, at 24 hrs post transfection, HEK293T cells were washed once with PBS and collected in 500 μL homogenization buffer (150 mM KCl, 20 mM HEPES pH 7.4, 2 mM EDTA) containing protease inhibitors and passed 30 times through a 27G-needle. The lysate was centrifuged at 2000 x g for 5 min at 4°C to remove the nuclear fraction. The supernatant was subsequently centrifuged at 100,000 x g for 1 h at 4°C to pellet the membrane fraction. The supernatant was removed (cytoplasmic fraction) and the pellet (membrane fraction) was resuspended in 80 μL of homogenization buffer. Twenty micrograms of cytoplasmic and membrane fractions were separated by SDS-PAGE and blotted with anti-GFP rabbit serum (Life Technologies). Rabbit anti-calnexin [clone ab13505] and mouse anti-GAPDH [clone 6C5] antibodies (Abcam) were used as markers of the membrane and cytosol fractions, respectively.

### Ionomycin treatment

Transfected HeLa cells were washed with PBS and incubated in 100 μL of either phosphate buffered saline (PBS) or Krebs-Ringer solution (120 mM NaCl, 4.7 mM KCl, 1.1 mM CaCl_2_, 0.7 mM MgSO_4_, 10 mM glucose, 10 mM Na-HEPES, pH 7.4). Ionomycin (Sigma Aldrich) was added to a final concentration of 5 μM and cells were incubated for 10 min. EGTA was added to a final concentration of 2 mM and cells were incubated for 10 min. Cells were then infected with *R*. *typhi* (described above) or fixed and stained as described above.

### Protein pull-down

RalF_Rt_ was cloned into the pTrcHisA vector (Life Technologies, see S1 Table for primer sequences) and transformed into Top10 *E*. *coli* cells (Life Technologies). Protein expression was induced with 1 mM IPTG overnight at 30°C. *E*. *coli* were lysed using Pierce Lysis Buffer in the presence of HALT Protease Inhibitors (Thermo Scientific) and imidazole added to a final concentration of 10 mM. Lysates were sonicated three times for 20 sec each using setting 6 of a Sonic Dismembranator (Fisher Scientific). mRFP-Arf5 and –Arf6 were expressed in HEK293T cells as described above. HEK293T cells were lysed using Pierce Lysis Buffer in the presence of HALT Protease Inhibitors (Thermo Scientific) and imidazole added to a final concentration of 10 mM.

Pull-down assays were performed using the Pierce Pull-Down PolyHis Protein:Protein Interaction kit according to manufacture’s protocol. Briefly, HisPur Cobalt Resin was incubated with rHis-RalF_Rt_ *E*. *coli* lysate or buffer alone for 1 hr. The resin was washed 5 times and then incubated with either mRFP-Arf5 or -Arf6 HEK293T lysate for 2 hr. Resin was again washed 5 times and bound proteins eluted with 290 mM imidazole elution buffer. Eluted proteins and 10% of the input protein were analyzed by protein immunoblot using the primary antibodies rabbit anti-RalF_Rt_, rabbit anti-Arf5 (1:1000, Thermo Scientific, PA5-31432) and rabbit anti-Arf6 (1:1000, Thermo Scientific, PA1-093) and the secondary antibody HRP anti-rabbit IgG (1:2000, BioLegend, clone 6B9G9).

### Gene knockdown

Negative and MISSION siRNAs against human Arf5 (SASI_Hs01_00024789) and Arf6 (SASI_Hs02_0033275) were obtained from Sigma Aldrich. All siRNA knockdowns were performed in HeLa cells using Lipofectamine 2000 (Life Technologies). Cells were used 24 hrs post transfection. Knockdowns were verified by western blot analysis using 1:1000 dilution of primary antibodies rabbit anti-Arf5 or anti-Arf6 (Thermo Scientific). As a loading control, membranes were re-probed with rabbit anti-GAPDH antibody (1:1000, Abcam).

### Data analysis

Graphs show the mean ± SD of three independent experiments; 100 cells were counted for each condition in every experiment. Statistical analyses were performed using two-tailed equal variance Student’s t-test.

## Supporting Information

S1 Fig Qualification of anti-ralF antibody. (PDF)

S2 Fig Comparative analysis of the Sec7 domain of RalF proteins from species of *Legionella* and *Rickettsia*. (PDF)

S3 Fig Comparative analysis of the Sec7-capping domain of RalF proteins from species of *Legionella* and *Rickettsia*. (PDF)

S4 Fig Characteristics of the extended C-terminal domain of *Rickettsia* RalF proteins. (PDF)

S5 Fig Genome synteny analysis across the *ralF*_*R*_ loci from select *Rickettsia* genomes. (PDF)

S6 Fig Densitometry analysis of membrane fractionation. (PDF)

S7 Fig Subcellular localization of rickettsial RalF proteins to Golgi apparatus. (PDF)

S8 Fig Quantification of rickettsial RalF proteins co-localization with plasma membrane, endoplasmic reticulum and Golgi apparatus. (PDF)

S9 Fig Arf1 does not co-localize with RalF_R_. (PDF)

S1 Table Primers used for this study. (PDF)
