# Supplementary material for: Which Way In? The RalF Arf-GEF Orchestrates Rickettsia Host Cell Invasion
Source: PLoS Pathog. 2015 Aug 20;11(8):e1005115. doi: 10.1371/journal.ppat.1005115 (PMC4546372; doi:10.1371/journal.ppat.1005115)
Supplement: S1 Fig — (PDF) [file ppat.1005115.s001.pdf]

S1 Fig. Qualification of anti-RalF$_{Rt}$ antibody. Expression of endogenous RalF$_{Rt}$. Immunoblot with (left) rabbit pre-immune serum, (center) Melon Gel IgG (Thermo Scientific) purified anti-RalF$_{Rt}$ antibody or (right) affinity purified anti-RalF$_{Rt}$ antibody. M, marker; Lanes 2 and 4, *R. typhi* 48 hr infected Vero76 whole lysate; Lanes 3 and 5, Vero76 whole lysate; Lane 6, *R. typhi* 48 hr infected HeLa lysate; Lane 7, HeLa whole lysate. Arrow indicates RalF$_{Rt}$ predicted size.

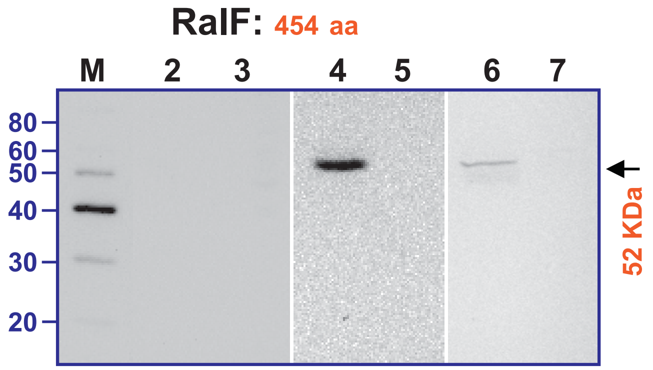

RalF: 454 aa
M
2
3
4
5
6
7
80
60
50
40
30
20
52 KDa
