# Supplementary material for: Which Way In? The RalF Arf-GEF Orchestrates Rickettsia Host Cell Invasion
Source: PLoS Pathog. 2015 Aug 20;11(8):e1005115. doi: 10.1371/journal.ppat.1005115 (PMC4546372; doi:10.1371/journal.ppat.1005115)
Supplement: S2 Fig — (PDF) [file ppat.1005115.s002.pdf]

S2 Fig. Comparative analysis of the Sec7 domain of RalF proteins from species of *Legionella* and *Rickettsia*. (A) Comparison of the crystal structure of *Legionella pneumophila* RalF (PDB 4C7P) [1] with the predicted structure of *R. bellii* str. RML369-C RalF (RBE_0868). Modeling done with Phyre2 [2]. The delineation of the Sec7 domain (S7D, red) and Sec7-capping domain (SCD, green) is shown, with an approximation of the active site Glu (asterisk). Like *R. typhi* (**Fig. 2**), *R. bellii* and other full length $RalF_R$ proteins contain an extended C-terminal domain relative to *L. pneumophila* RalF (see **S4 Fig.**). One distinguishing feature of RBE_0868 relative to all other RalF proteins is an insertion (AESKLQGIK) in the S7D, which is predicted to form small beta sheets between helices I and J (denoted with blue star). (B) Comparison of the S7D of *Legionella* and *Rickettsia* RalF proteins. Cladogram depicts a branching pattern recovered from an estimated phylogeny that included select eukaryotic Sec7-domain-containing proteins (e.g., ARNO, BRAG2, EFA6, etc.). Briefly, the Sec7 domains of RalF proteins and select eukaryotic Sec7-domain-containing proteins were aligned with MUSCLE (default parameters) [3], with a phylogeny estimated under maximum likelihood (LG substitution model) using RAxML v.8 [4]. A gamma model of rate heterogeneity was used with estimation of the proportion of invariable sites. Branch support was assessed with 1000 bootstrap pseudoreplications (shown in colored circles, which are described below the tree). The alignment shown for the S7D of RalF proteins was performed separately without the eukaryotic sequences, using MUSCLE (default parameters). The secondary structure of *L. pneumophila* RalF (PDB 4C7P) [1] is superimposed over the alignment. Conserved residues are highlighted yellow, with two highly conserved regions (Motif 1 and Motif 2) that together form the Sec7 active site boxed in red [5]. The active site Glu of Motif 1, which is essential for Arf recruitment to the *Legionella* containing vacuole [6], is further distinguished in red and noted with an asterisk. Within Motif 2, divergent residues of *R. bellii* RalF proteins are colored black. *Rickettsia* RalF proteins are distinguished with gray shading. NCBI GenBank accession numbers for all proteins as follows: *Legionella shakespeare*, WP_018578688; *L. moravica*, ACQ90228; *L. pneumophila* subsp. *pneumophila* str. Philadelphia 1, YP_095966; *L. pneumophila* str. Corby, YP_001250720; *L. pneumophila* str. Lens, YP_127255; *L. pneumophila* str. Paris, YP_124246; *L. pneumophila* str. Leg01/11, ERH43534; *L. longbeachae* str. NSW150, YP_003454872; *L. sainthelensi*, WP_027272333; *Rickettsia bellii* str. RML369-C, YP_538038; *R. bellii* str. OSU 85-389, YP_001495923; *R. felis* str. URRWXCal2, YP_246601; *R. akari* str. Hartford, YP_001493357; *R. australis* str. Cutlack, YP_005415187; *R. typhi* str. Wilmington, YP_067323; *R. prowazekii* str. GvV257, YP_005404890; *R. prowazekii* str. Madrid E, NP_220757; *R. prowazekii* str. Chernikova, YP_005405467. (C) Comparison of the sequences

within Motif 1 and Motif 2 of the Sec7 active site across *R. bellii* RalF and select eukaryotic Arf-GEFs. Conserved residues are highlighted yellow, with hydrophobic residues colored orange. Two residues within Motif 2 (colored black) are divergent in *R. bellii* relative to all eukaryotic Arf-GEFs: Ser at position one shifts the polarity of the active site, while Gly at position seven presents a shorter side chain. NCBI GenBank accession numbers for eukaryotic Arf-GEFs as follows: protein transport protein SEC7 of *Saccharomyces cerevisiae* S288c (P11075.2); EFA6 ARF GEF of *Caenorhabditis elegans* (ABQ42569); exchange factor for Arf 6 ortholog, isoform I of *Drosophila melanogaster* (AAO41590.3); EFA6A of *Homo sapiens* (NP_001257894); EFA6B of *Homo sapiens* (Q8NDX1.2); EFA6C of *Homo sapiens* (NP_115665); EFA6D of *Homo sapiens* (Q2PFD7.2); EFA6E of *Homo sapiens* (NP_055684.3); ARNO of *Homo sapiens* (CAA68084).

References

1. Folly-Klan M, Alix E, Stalder D, Ray P, Duarte L V., Delprato A, et al. A Novel Membrane Sensor Controls the Localization and ArfGEF Activity of Bacterial RalF. PLoS Pathog. 2013;9. doi:10.1371/journal.ppat.1003747

2. Kelley LA, Sternberg MJE. Protein structure prediction on the Web: a case study using the Phyre server. Nat Protoc. 2009;4: 363–371. doi:10.1038/nprot.2009.2

3. Edgar RC. MUSCLE: Multiple sequence alignment with high accuracy and high throughput. Nucleic Acids Res. 2004;32: 1792–1797. doi:10.1093/nar/gkh340

4. Stamatakis A. RAxML version 8: A tool for phylogenetic analysis and post-analysis of large phylogenies. Bioinformatics. 2014;30: 1312–1313. doi:10.1093/bioinformatics/btu033

5. Derrien V, Couillault C, Franco M, Martineau S, Montcourrier P, Houlgatte R, et al. A conserved C-terminal domain of EFA6-family ARF6-guanine nucleotide exchange factors induces lengthening of microvilli-like membrane protrusions. J Cell Sci. 2002;115: 2867–79. Available: http://www.ncbi.nlm.nih.gov/pubmed/12082148

6. Alix E, Chesnel L, Bowzard BJ, Tucker AM, Delprato A, Cherfils J, et al. The Capping Domain in RalF Regulates Effector Functions. PLoS Pathog. 2012;8. doi:10.1371/journal.ppat.1003012

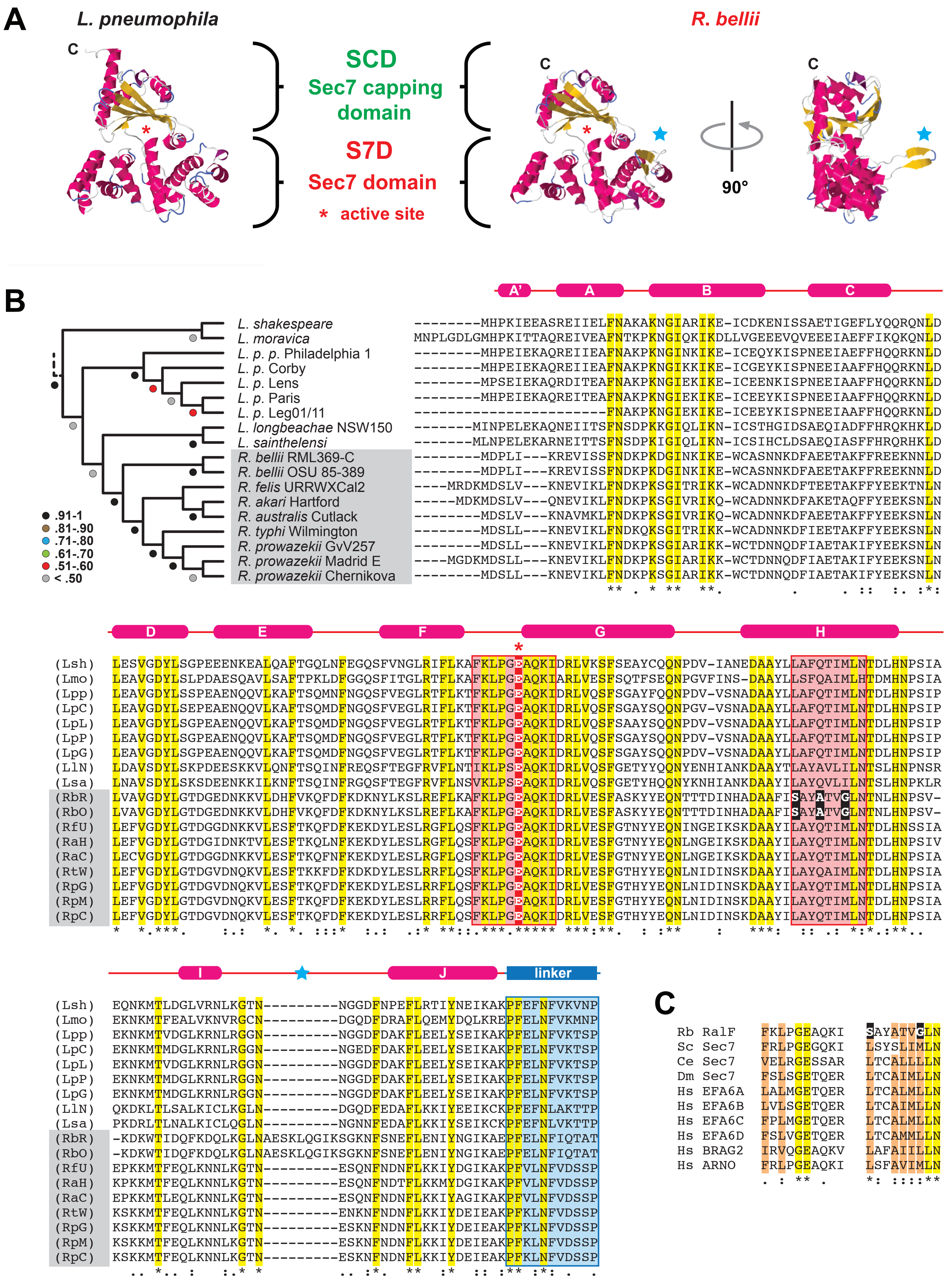
A
L. pneumophila
R. bellii
C
SCD
Sec7 capping domain
S7D
Sec7 domain
* active site
90°
B
A'
A
B
C
L. shakespeare --------MHPKIEEASREIIELFNAKAKKNGIARIKE-ICDKENISSAETIGEFLYQQRQNLD
L. moravica MNPLGDLGMHPKITTAQREIVEAFNTKPKNGIQKIKDLLVGEEEVQVEEEIAEFFIKQKQNLD
L. p. p. Philadelphia 1 --------MHPEIEKAQREIIEAFNAKPKNGINKIKE-ICEQYKISPNEEIAEFFHQQRKNLD
L. p. Corby --------MHPEIEKAQREIIEAFNAKPKNGIKKIKE-ICGEYKISPNEEIAAFFHQQRKNLD
L. p. Lens --------MPSEIEKAQRDITEAFNAKPKNGIEKIKE-ICEENKISPNEEIAAFFHQQRKNLD
L. p. Paris --------MHPEIEKAQREITEAFNAKPKNGIEKIKE-ICEEYKISPNEEIAAFFHQQRKNLD
L. p. Leg01/11 ------------------------FNAKPKNGIEKIKE-ICEEYKISPNEEIAAFFHQQRKNLD
L. longbeachae NSW150 -------MINPELEKAQNEIITSFNSDPKKGIQLIKN-ICSTHGIDSAEQIADFFHRQKHKLD
L. sainthelensi -------MLNPELEKARNEITTSFNSDPKIGIQLIKN-ICSIHCLDSAEQIASFFHRQRHKLD
R. bellii RML369-C --------MDPLI---KREVISSFNDKPKDGINKIKE-WCASNNKDFAEETAKFFREEKNNLD
R. bellii OSU 85-389 --------MDPLI---KREVISSFNDKPKDGINKIKE-WCASNNKDFAEETAKFFREEKNNLD
R. felis URRWXCal2 ----MRDKMDSLV---KNEVIKLFNDKPKSGITRIKK-WCADNNKDFTEETAKFFYEEKTNLN
R. akari Hartford -----MDKMDSLV---QNEVIKLFNDKPKSGIVRIKQ-WCADNNKDFAKETAQFFYEEKSNLN
R. australis Cutlack --------MDSLV---KNAVMKLFNDKPKSGIVRIKQ-WCADNNKDFAEETAKFFYEEKSNLN
R. typhi Wilmington --------MDSLL---KNEVIKLFNDKQKSGITRIKK-WCADNNKDFIEETAKFFYEEKSNLN
R. prowazekii GvV257 --------MDSLL---KNEVIKLFNDKPKSGIARIKK-WCTDNNQDFIAETAKIFYEEKSNLN
R. prowazekii Madrid E ----MGDKMDSLL---KNEVIKLFNDKPKSGIARIKK-WCTDNNQDFIAETAKIFYEEKSNLN
R. prowazekii Chernikova --------MDSLL---KNEVIKLFNDKPKSGIARIKK-WCTDNNQDFIAETAKIFYEEKSNLN
** . * ** **. . . :: :. :*:
.91-1
.81-.90
.71-.80
.61-.70
.51-.60
< .50
D
E
F
G
H
*
(Lsh) LESVGDYLSGPEEENKEALQAFTGQLNFEGQSFVNGLRIFLKAFKLPGEAQKIDRLVKSFSEAYCQQNPDV-IANEDAAYLLAFQTIMLNTDLHNPSIA
(Lmo) LEAVGDYLSLPDAESQAVLSAFTPKLDFGGQSFITGLRTFLKAFKLPGEAQKIARLVESFSQTFSEQNPGVFINS-DAAYLLSFQAIMLHTDMHNPSIA
(Lpp) LEAVGDYLSSPEAENQQVLKAFTSQMNFNGQSFVEGLRTFLKTFKLPGEAQKIDRLVQSFSGAYFQQNPDV-VSNADAAYLLAFQTIMLNTDLHNPSIP
(LpC) LEAVGDYLSEPEAENQQVLKAFTSQMDFNGQSFVEGLRIFLKTFKLPGEAQKIDRLVQSFSGAYSQQNPGV-VSNADAAYLLAFQTIMLNTDLHNPSIP
(LpL) LEAVGDYLSGPEAENQQVLKAFTSQMDFNGQSFVEGLRTFLKTFKLPGEAQKIDRLVQSFSAAYSQQNPDV-VSNADAAYLLAFQTIMLNTDLHNPSIP
(LpP) LEAVGDYLSGPEAENQQVLKAFTSQMDFNGQSFVEGLRTFLKTFKLPGEAQKIDRLVQSFSGAYSQQNPDV-VSNADAAYLLAFQTIMLNTDLHNPSIP
(LpG) LEAVGDYLSGPEAENQQVLKAFTSQMDFNGQSFVEGLRTFLKTFKLPGEAQKIDRLVQSFSGAYSQQNPDV-VSNADAAYLLAFQTIMLNTDLHNPSIP
(LlN) LDAVSDYLSKPDEESKKVLQNFTSQINFREQSFTEGFRVFLNTIKLPSEAQKIDRLVQSFGETYYQQNYENHIANKDAAYTLAYAVLILNTSLHNPNSR
(Lsa) LNAVSDYLSKSDEENKKILKNFTSQINFRGQSFTEGFRVFLNSVKLPSEAQKIDRLVQSFGETYHQQNYKNHIANKDAAYILAYQVLILNTSLHNPKLR
(RbR) LVAVGDYLGTDGEDNKKVLDHFVKQFDFKDKNYLKSLREFLKAFKLPGEAQKIDRLVESFASKYYEQNTTTDINHADAAFISAYATVGLNTNLHNPSV-
(RbO) LVAVGDYLGTDGEDNKKVLDHFVKQFDFKDKNYLKSLREFLKAFKLPGEAQKIDRLVESFASKYYEQNTTTDINHADAAFISAYATVGLNTNLHNPSV-
(RfU) LEFVGDYLGTDGGDNKKVLESFTKQFDFKEKDYLESLRGFLQSFKLPGEAQKIDRLVESFGTNYYEQNINGEIKSKDAAYILAYQTIMLNTDLHNSSIA
(RaH) LEFVGDYLGTDGIDNKTVLESFTKQLNFKEKDYLESLRGFLQSFKLPGEAQKIDRLVESFGTNYYEQNLNGEIKSKDAAYILAYQTIMLNTDLHNPSIV
(RaC) LECVGDYLGTDGGDNKKVLESFTKQFNFKEKDYLESLRGFLQSFKLPGEAQKIDRLVESFGTNYYEQNLNGEIKSKDAAYILAYQTIMLNTDLHNPSIA
(RtW) LEFVGDYLGTDGVDNQKVLESFTKKFDFKDKDYLESLRRFLQSFKLPGEAQKIDRLVESFGTHYYEQNLNIDINSKDAAYILAYQAIMLNTDLHNPSIA
(RpG) LEFVGDYLGTDGVDNQKVLESFTKQFDFKEKDYLESLRRFLQSFKLPGEAQKIDRLVESFGTHYYEQNLNIDINSKDAAYILAYQTIMLNTDLHNPSIA
(RpM) LEFVGDYLGTDGVDNQKVLESFTKQFDFKEKDYLESLRRFLQSFKLPGEAQKIDRLVESFGTHYYEQNLNIDINSKDAAYILAYQTIMLNTDLHNPSIA
(RpC) LEFVGDYLGTDGVDNQKVLESFTKQFDFKEKDYLESLRRFLQSFKLPGEAQKIDRLVESFGTHYYEQNLNIDINSKDAAYILAYQTIMLNTDLHNPSIA
* *.***. :.: *. *. :::* :.: .:* **::.***.***** ***:**. : :** : ***: :: .: *:*.:**..
I
J
linker
(Lsh) EQNKMTLDGLVRNLKGTN---------NGGDFNPEFLRTIYNEIKAKPFEFNFVKVNP
(Lmo) EKNKMTFEALVKNVRGCN---------DGQDFDRAFLQEMYDQLKREPFELNFVKMNP
(Lpp) EKNKMTVDGLKRNLRGGN---------NGGDFDAKFLEELYSEIKAKPFELNFVKTSP
(LpC) EKNKMTMDGLKRNLRGGN---------NGEDFDAKFLEELYSEIKAKPFELNFVKTSP
(LpL) EKNKMTVDGLKRNLRGGN---------NGGDFDAKFLEELYSEIKAKPFELNFVKTSP
(LpP) EKNKMTMDGLKRNLRGGN---------NGGDFDAKFLEELYSEIKAKPFELNFVKTSP
(LpG) EKNKMTMDGLKRNLRGGN---------NGGDFDAKFLEELYSEIKAKPFELNFVKTSP
(LlN) QKDKLTLSALKICLKGLN---------DGQDFEDAFLKKIYEEIKCKPFEFNLAKTTP
(Lsa) PKDRLTLNALKICLQGLN---------NGNNFEDAFLKKIYSEIKCKPFEFNLVKTTP
(RbR) -KDKWTIDQFKDQLKGLNAESKLQGIKSGKNFSNEFLENIYNGIKAEPFELNFIQTAT
(RbO) -KDKWTIDQFKDQLKGLNAESKLQGIKSGKNFSNEFLENIYNGIKAEPFELNFIQTAT
(RfU) EPKKMTFEQLKNNLKGTN---------ESQNFNDNFLKKIYDGIKAKPFVLNFVDSSP
(RaH) KPKKMTFEQLKNNLKGTN---------ESQNFNDTFLKKMYDGIKAKPFVLNFVDSSP
(RaC) EPKKMTLEQLKNNLKGTN---------ESQNFNDNFLKKIYAGIKAKPFVLNFVDSSP
(RtW) KSKKMTFEQLKNNLKGTN---------ENKNFNDNFLKKIYDEIEAKPFKLNFVDSSP
(RpG) KSKKMTFEQLKNNLKGTN---------ESKNFNDNFLKKIYDEIEAKPFKLNFVDSSP
(RpM) KSKKMTFEQLKNNLKGTN---------ESKNFNDNFLKKIYDEIEAKPFKLNFVDSSP
(RpC) KSKKMTFEQLKNNLKGTN---------ESKNFNDNFLKKIYDEIEAKPFKLNFVDSSP
.. *.. : :.* * .. :*. ** :* :: :** :*: . .
C
Rb RalF FKLPGEAQKI SAYATVGLN
Sc Sec7 FRLPGEGQKI LSYSLIMLN
Ce Sec7 VELRGESSAR LTCALLLLN
Dm Sec7 FSLSGETQER LTCAIMLLN
Hs EFA6A LALMGETQER LTCALMLLN
Hs EFA6B LVLSGETQER LTCAIMLLN
Hs EFA6C FPLMGETQER LTCALMLLN
Hs EFA6D FSLVGETQER LTCAMMLLN
Hs BRAG2 IRVQGEAQKV LAFAIILLN
Hs ARNO FRLPGEAQKI LSFAVIMLN
. : ** . *: ::::**
