# Supplementary material for: Which Way In? The RalF Arf-GEF Orchestrates Rickettsia Host Cell Invasion
Source: PLoS Pathog. 2015 Aug 20;11(8):e1005115. doi: 10.1371/journal.ppat.1005115 (PMC4546372; doi:10.1371/journal.ppat.1005115)
Supplement: S3 Fig — (PDF) [file ppat.1005115.s003.pdf]

S3 Fig. Comparative analysis of the Sec7-capping domain of RalF proteins from species of *Legionella* and *Rickettsia*.

(A) Comparison of the crystal structure of *Legionella pneumophila* RalF (PDB 4C7P) [1] with the predicted structure of *R. typhi* RalF (RT0362). Modeling done with Phyre2 [2]. The delineation of the Sec7 domain (S7D, red) and Sec7-capping domain (SCD, green) is shown, with an approximation of the active site Glu (asterisk). The structural model presented in Fig. 2**A** does not include the terminal helix of the SCD, helix P. The alternative model presented here, wherein the 'NIR' insertion is shifted to align to 'NQK' of *L. pneumophila*, results in a 14 aa helix (DLKSKYDNIRNAKQ) that is still truncated relative to the 18 aa helix P of $RalF_L$. While a plausible model, the location of the 'NIR' insertion in our multiple sequence alignment is strongly supported (panel B). Two lines of evidence support a truncated helix P in $RalF_R$ proteins. First, modeling of *R. bellii* RalF to $RalF_L$, which lacks the 'NIR' insertion, still results in a truncated helix P, QDLENYYNNPEQ, (**S2 Fig.**). Second, the recently solved LpRpRalF chimeric structure (*L. pneumophila* S7D fused to *R. prowazekii* SCD, PDB 4D7Q) yielded a truncated helix P consistent with our modeling for *R. bellii* [3]. As the extended C-terminal domain was proteolyzed and not included in the LpRpRalF structure, it is likely that all $RalF_R$ proteins contain a truncated helix P that possibly diverges from $RalF_R$ proteins to elaborate the extended C-terminal domain. Another distinguishing factor of most $RalF_R$ proteins, except *R. bellii*, is an insertion (REDGKQP in *R. typhi*) located between beta strand 5 and helix O. The functional significance of this expanded loop, particularly its possible contacts made with the S7D, remains unknown. It is noteworthy that this loop is expanded in other $RalF_L$ proteins. (B) Comparison of the SCD of *Legionella* and *Rickettsia* RalF proteins. Cladogram depicts the estimated phylogeny described in **S2 Fig.** The alignment shown for the SCD of RalF proteins was performed using MUSCLE (default parameters) [4]. The secondary structure of *L. pneumophila* RalF (PDB 4C7P) [1] is superimposed over the alignment. Conserved residues are highlighted yellow. Aromatic clusters comprising the membrane sensor region are enclosed in green boxes, with aromatic residues (Phe, Trp, Tyr) green and positively charged residues (Arg, His, Lys) blue. Following previous mutagenesis analysis of the SCD [1]: **#**, residues in $RalF_L$ (*L. pneumophila*) permuted to the corresponding $RalF_R$ (*R. prowazekii*) and vice versa; **^**, residues in $RalF_L$ (*L. pneumophila*) permuted to the corresponding $RalF_R$ (*R. prowazekii*) but not reciprocated. Three regions wherein the *R. bellii* RalF SCD is more characteristic of $RalF_L$ proteins than $RalF_R$ proteins are within red boxes. The highly conserved KATY motif, which contacts the Sec7 domain remote from the active site and is thought to function as a hinge for the conformational change that activates RalF [1], is colored black. *Rickettsia* RalF proteins are

distinguished with gray shading. NCBI GenBank accession numbers for all proteins are listed in S2 Fig. C) Emphasis on the SCD lipid sensor. The two aromatic clusters comprising the SCD lipid sensor are merged together, with highlighting as follows: five conserved residues, yellow; two residues comprised solely of positively charged residues, blue. Additionally, four residues conserved in positive charge (all Lys) for *Rickettsia* spp. are also highlighted blue and denoted with a red arrow over the alignment. One other residue strongly conserved in positively charged residues, but replaced with a Thr in all strains of *L. pneumophila* and *R. australis* str. Cutlack, is denoted above the alignment by a gray arrow. Although no positions within the lipid sensor are conserved in aromatic residues across all RalF proteins, $RalF_L$ proteins are generally more aromatic. The lipid sensors of $RalF_R$ proteins encompass a markedly higher ratio of positively charged residues, consistent with their previously demonstrated affinity for negatively-charged lipids, particularly cardiolipin and the phosphoinositides $PI(4,5)P_2$ and $PI(3,4,5)P_3$ [5]. It is noteworthy to consider the drastic divergent biochemical profiles of *L. pneumophila* and *R. prowazekii* lipid sensors in light of the lower levels of aromatic residues and higher levels of positively charged residues for non-*pneumophila* species of *Legionella*.

References

1. Folly-Klan M, Alix E, Stalder D, Ray P, Duarte L V., Delprato A, et al. A Novel Membrane Sensor Controls the Localization and ArfGEF Activity of Bacterial RalF. PLoS Pathog. 2013;9. doi:10.1371/journal.ppat.1003747

2. Kelley LA, Sternberg MJE. Protein structure prediction on the Web: a case study using the Phyre server. Nat Protoc. 2009;4: 363–371. doi:10.1038/nprot.2009.2

3. Folly-Klan M, Sancerne B, Alix E, Roy CR, Cherfils J, Campanacci V. On the use of *Legionella*/*Rickettsia* chimeras to investigate the structure and regulation of *Rickettsia* effector RalF. J Struct Biol. 2014; doi:10.1016/j.jsb.2014.12.001

4. Edgar RC. MUSCLE: Multiple sequence alignment with high accuracy and high throughput. Nucleic Acids Res. 2004;32: 1792–1797. doi:10.1093/nar/gkh340

5. Alix E, Chesnel L, Bowzard BJ, Tucker AM, Delprato A, Cherfils J, et al. The Capping Domain in RalF Regulates Effector Functions. PLoS Pathog. 2012;8. doi:10.1371/journal.ppat.1003012

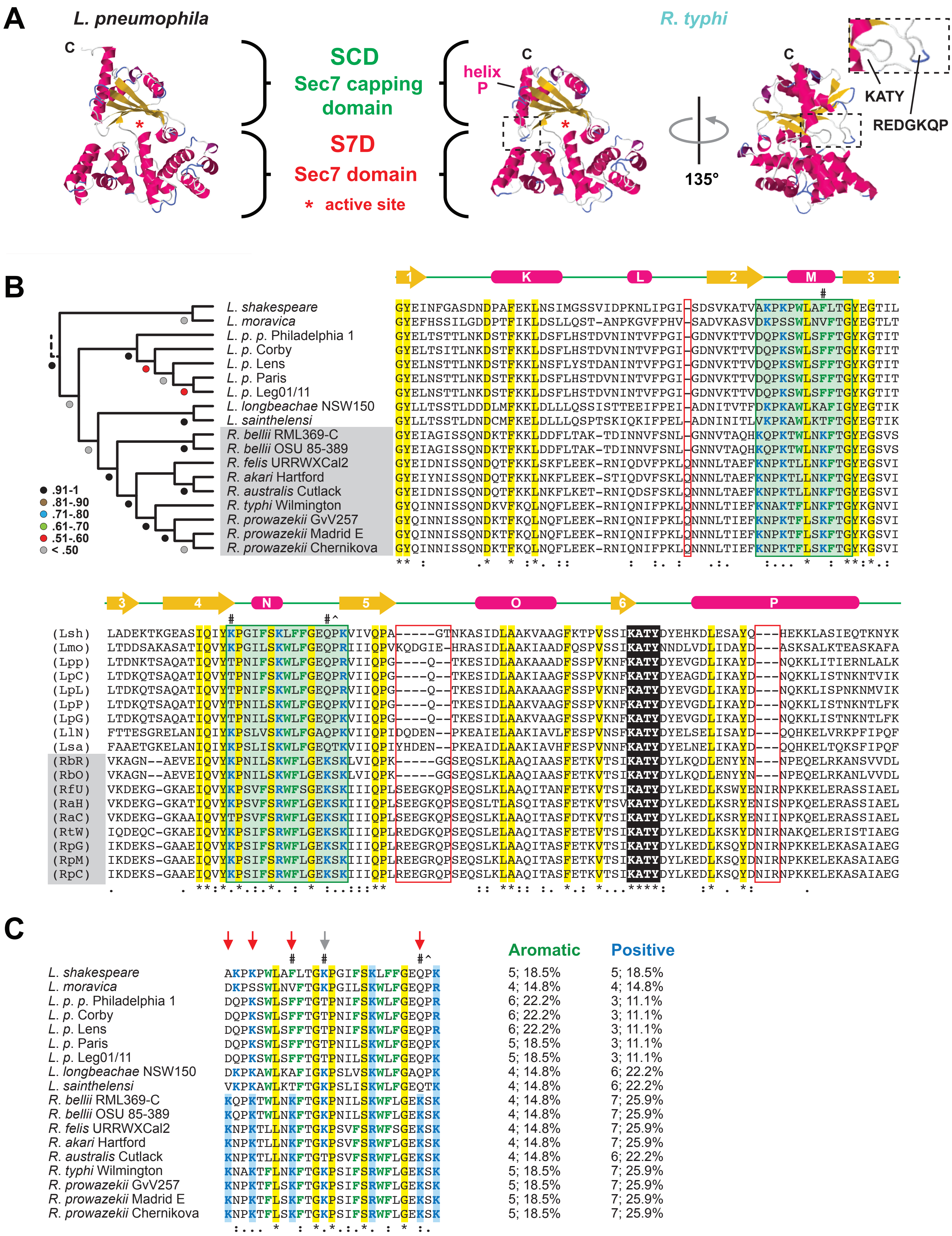

A
L. pneumophila
R. typhi
SCD
Sec7 capping domain
S7D
Sec7 domain
* active site
helix P
KATY
REDGKQP
135°
C
B
L. shakespeare
L. moravica
L. p. p. Philadelphia 1
L. p. Corby
L. p. Lens
L. p. Paris
L. p. Leg01/11
L. longbeachae NSW150
L. sainthelensi
R. bellii RML369-C
R. bellii OSU 85-389
R. felis URRWXCal2
R. akari Hartford
R. australis Cutlack
R. typhi Wilmington
R. prowazekii GvV257
R. prowazekii Madrid E
R. prowazekii Chernikova
.91-1
.81-.90
.71-.80
.61-.70
.51-.60
< .50
1 K L 2 M 3
3 4 N 5 O 6 P
(Lsh) (Lmo) (Lpp) (LpC) (LpL) (LpP) (LpG) (LlN) (Lsa) (RbR) (RbO) (RfU) (RaH) (RaC) (RtW) (RpG) (RpM) (RpC)
C
Aromatic
Positive
L. shakespeare AKPKPWLAFLTGKPGIFSKLFFGEQPK 5; 18.5% 5; 18.5%
L. moravica DKPSSWLNVFTGKPGILSKWLFGEQPR 4; 14.8% 4; 14.8%
L. p. p. Philadelphia 1 DQPKSWLSFFTGTPNIFSKWLFGEQPR 6; 22.2% 3; 11.1%
L. p. Corby DQPKSWLSFFTGTPNIFSKWLFGEQPR 6; 22.2% 3; 11.1%
L. p. Lens DQPKSWLSFFTGTPNIFSKWLFGEQPR 6; 22.2% 3; 11.1%
L. p. Paris DQPKSWLSFFTGTPNILSKWLFGEQPK 5; 18.5% 3; 11.1%
L. p. Leg01/11 DQPKSWLSFFTGTPNILSKWLFGEQPK 5; 18.5% 3; 11.1%
L. longbeachae NSW150 DKPKAWLKAFIGKPSLVSKWLFGAQPK 4; 14.8% 6; 22.2%
L. sainthelensi VKPKAWLKTFTGKPSLISKWLFGEQTK 4; 14.8% 6; 22.2%
R. bellii RML369-C KQPKTWLNKFTGKPNILSKWFLGEKSK 4; 14.8% 7; 25.9%
R. bellii OSU 85-389 KQPKTWLNKFTGKPNILSKWFLGEKSK 4; 14.8% 7; 25.9%
R. felis URRWXCal2 KNPKTLLNKFTGKPSVFSRWFSGEKSK 4; 14.8% 7; 25.9%
R. akari Hartford KNPKTLLNKFTGKPSVFSRWFLGEKSK 4; 14.8% 7; 25.9%
R. australis Cutlack KNPKTLLNKFTGTPSVFSRWFLGEKSK 4; 14.8% 6; 22.2%
R. typhi Wilmington KNAKTFLNKFTGKPSIFSRWFLGEKSK 5; 18.5% 7; 25.9%
R. prowazekii GvV257 KNPKTFLSKFTGKPSIFSRWFLGEKSK 5; 18.5% 7; 25.9%
R. prowazekii Madrid E KNPKTFLSKFTGKPSIFSRWFLGEKSK 5; 18.5% 7; 25.9%
R. prowazekii Chernikova KNPKTFLSKFTGKPSIFSRWFLGEKSK 5; 18.5% 7; 25.9%
