# Supplementary material for: Which Way In? The RalF Arf-GEF Orchestrates Rickettsia Host Cell Invasion
Source: PLoS Pathog. 2015 Aug 20;11(8):e1005115. doi: 10.1371/journal.ppat.1005115 (PMC4546372; doi:10.1371/journal.ppat.1005115)
Supplement: S4 Fig — (PDF) [file ppat.1005115.s004.pdf]

S4 Fig. Characteristics of the extended C-terminal domain of *Rickettsia* RalF proteins.

All alignments were performed using MUSCLE (default parameters) [1]. (A) Sequence alignment of the variable sequence with Pro-rich region (VPR) and T4SS signal sequence (T4S) across select *Rickettsia* species. *Rickettsia* species are organized based on recent estimated phylogeny [2], with classification scheme as follows: red, ancestral group (AG); blue, transitional group (TRG); aquamarine, typhus group (TG); and brown, spotted fever group (SFG). *R. helvetica* and *R. tamurae* (black) are unclassified following recent recommendations [3]. Red circles denote full-length $RalF_{R}$ proteins, with NCBI GenBank accession numbers for these proteins provided in **S2 Fig.** Accession numbers for all other proteins as follows: *Rickettsia canadensis* str. McKiel, YP_001492434; *R. tamurae*, WP_032139614; *R. helvetica*, WP_010423297; *R. akari* str. Hartford, YP_001493241; REIS (*Rickettsia* endosymbiont of *Ixodes scapularis*), EER21763; *Rickettsia* sp. ISO7, KDO03638; *R. monacensis str.* IrR/Munich, CDI29630; "*Candidatus* Rickettsia amblyommii" str. GAT-30V, YP_005365253; *R. aeschlimannii*, WP_032074125; *R. rhipicephali* str. 3-7-female6-CWPP, YP_005390352; *R. montanensis* str. OSU 85-930, YP_005392197; "*Candidatus* Rickettsia gravesii", WP_017443103; *R. heilongjiangensis* str. 054, YP_004764228; *R. japonica*, WP_024704516; *R. honei*, WP_032850143 and WP_016916690; *R. philipii* str. 364D, YP_005300602; *R. rickettsii* str. Brazil, YP_005292780 and YP_005292779; *R. slovaca* str. D-CWPP, YP_005426261, YP_005426262 and YP_005426263; *R. conorii* str. Malish 7, NP_360145 and NP_360146; *R. parkeri* str. Portsmouth, YP_005392764; *R. sibirica*, WP_026054964. Multiple accession numbers for several species denote chimeras comprised of multiple fragments corresponding to the full-length VPR sequences; green highlighting demarcates the boundaries of the individual ORFs within these chimeras, with green dots depicting missing sequence. For several proteins, misaligned terminal residues were stitched back to their respective ends (shown in red), though their homology to these positions is doubtful. Above the alignment, the blue bar depicts the VPR, and the orange bar depicts the T4S. Gray shading illustrates the large deletion within the VPR of RalF proteins from TG rickettsiae. Purple shading depicts the Pro-rich region with the VPR, with Pro residues colored purple. (B) Alignment of the T4S across full-length $RalF_{R}$ proteins. Conserved residues are highlighted yellow, with positively charged residues (Arg, His, Lys) colored blue. One conserved positively charged residue (-12 from the C-terminus) is highlighted blue. (C) The T4S does not affect $RalF_{Rt}$ localization. HeLa cells expressing YFP tagged $RalF_{RtCTD\Delta T4S}$ (green, described in **Fig. 2B**) were stained with Alexa Fluor 594 Phalloidin probe to label actin (red). DAPI (blue) is shown in the merged image. Cytoplasmic (C) and membrane (M) localization was confirmed via membrane fractionation of

HEK293T cells Lipofectamine 2000 transfected with the indicated plasmids followed by immunoblotting. Immunoblot: 1, primary Ab, rabbit anti-GFP (Life Technologies); 2, rabbit anti-Calnexin (Abcam); 3, mouse anti-GAPDH (Abcam). (D) Alignment of the C-terminal sequences across select $RalF_L$ proteins. NCBI GenBank accession numbers for all proteins are provided in **S2 Fig.** For *L. pneumophila* subsp. *pneumophila* str. Philadelphia 1, the Leu residue at position -3 (relative to the C-terminus) is within a black box. This L372 residue has been shown to be critical for RalF translocation via the *dot*/*icm* T4SS [4]. Unlike the C-terminal sequences of $RalF_R$ proteins (panel B), the C-terminal sequences of $RalF_L$ proteins are not conserved, with no other $RalF_L$ sequences harboring a Leu or other hydrophobic residue at position -3, and only some sequences harboring a hydrophobic residue(s) in the terminal five aa positions (colored orange). (E) The VPR sequences across select *Rickettsia* species contain a predicted coiled-coil motif. Conserved residues are highlighted yellow, with residues within predicted coiled-coil motifs colored green. A consensus coiled-coil motif is depicted at the top of the alignment. Red arrows demarcate the region of the VPR that is absent from TG rickettsiae RalF proteins. Coiled-coil motifs were predicted using the COILS server [5]. (F) Schema depicting the prototypical $RalF_R$ protein. See text for further details. Alignments provided for the S7D (**S2 Fig.**), SCD (**S3 Fig.**) and extended C-terminal domain (VPR and T4S) (panel A) provide the delineations of these discrete domains. (G) Breakdown of the amino acid composition within four discrete regions (S7D, SCD, VPR, T4S) for six full-length $RalF_R$ proteins. Within each region, amino acid frequencies were calculated and grouped into four major categories: non-polar/aliphatic (Ala, Gly, Ile, Leu, Met, Val), polar (Asn, Cys, Gln, Pro, Ser, Thr), aromatic (Phe, Trp, Tyr), positively charged (Arg, His, Lys), and negatively charged (Asp, Glu). While non-polar, proline was considered polar since it is typically solvent-exposed, despite having an aliphatic side chain.

References

1. Edgar RC. MUSCLE: Multiple sequence alignment with high accuracy and high throughput. Nucleic Acids Res. 2004;32: 1792–1797. doi:10.1093/nar/gkh340

2. Gillespie JJ, Kaur SJ, Rahman MS, Rennoll-Bankert K, Sears KT, Beier-Sexton M, et al. Secretome of obligate intracellular *Rickettsia*. FEMS Microbiol Rev. 2014; 1–44. doi:10.1111/1574-6976.12084

3. Driscoll T, Gillespie JJ, Nordberg EK, Azad AF, Sobral BW. Bacterial DNA sifted from the *Trichoplax adhaerens* (Animalia: Placozoa) genome project reveals a putative rickettsial endosymbiont. Genome Biol Evol. 2013;5: 621–45. doi:10.1093/gbe/evt036

4. Nagai H, Cambronne ED, Kagan JC, Amor JC, Kahn RA, Roy CR. A C-terminal translocation signal required for Dot/Icm-dependent delivery of the *Legionella* RalF protein to host cells. Proc Natl Acad Sci U S A. 2005;102: 826–831. doi:10.1073/pnas.0406239101

5. Lupas A, Van Dyke M, Stock J. Predicting coiled coils from protein sequences. Science. 1991;252: 1162–4. doi:10.1126/science.252.5009.1162

A

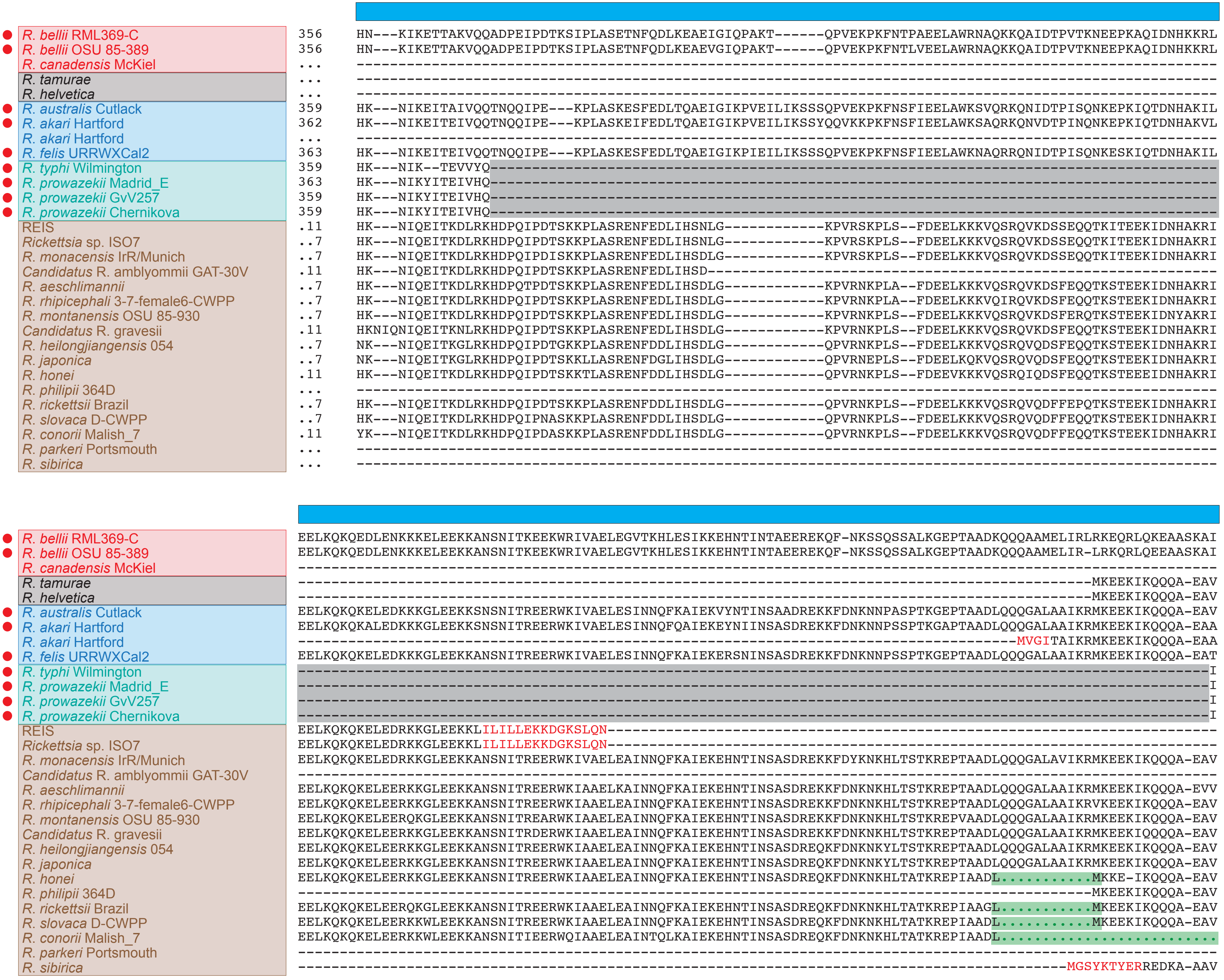

R. bellii RML369-C 356 HN---KIKETTAKVQQADPEIPDTKSIPLASETNFQDLKEAEIGIQPAKT------QPVEKPKFNTPAEELAWRNAQKKQAIDTPVTKNEEPKAQIDNHKKRL
R. bellii OSU 85-389 356 HN---KIKETTAKVQQADPEIPDTKSIPLASETNFQDLKEAEVGIQPAKT------QPVEKPKFNTLVEELAWRNAQKKQAIDTPVTKNEEPKAQIDNHKKRL
R. canadensis McKiel ... --
R. tamurae ... --
R. helvetica ... --
R. australis Cutlack 359 HK---NIKEITAIVQQTNQQIPE---KPLASKESFEDLTQAEIGIKPVEILIKSSSQPVEKPKFNSFIEELAWKSVQRKQNIDTPISQNKEPKIQTDNHAKIL
R. akari Hartford 362 HK---NIKEITEIVQQTNQQIPE---KPLASKEIFEDLTQAEIGIKPVEILIKSSYQQVKKPKFNSFIEELAWKSAQRKQNVDTPINQNKEPKIQTDNHAKVL
R. akari Hartford ... --
R. felis URRWXCal2 363 HK---NIKEITEIVQQTNQQIPE---KPLASKESFEDLTQAEIGIKPIEILIKSSSQPVEKPKFNSFIEELAWKNAQRRQNIDTPISQNKESKIQTDNHAKIL
R. typhi Wilmington 359 HK---NIK--TEVVYQ--
R. prowazekii Madrid_E 363 HK---NIKYITEIVHQ--
R. prowazekii GvV257 359 HK---NIKYITEIVHQ--
R. prowazekii Chernikova 359 HK---NIKYITEIVHQ--
REIS .11 HK---NIQEITKDLRKHDPQIPDTSKKPLASRENFEDLIHSNLG------------KPVRSKPLS--FDEELKKKVQSRQVKDSSEQQTKITEEKIDNHAKRI
Rickettsia sp. ISO7 ..7 HK---NIQEITKDLRKHDPQIPDTSKKPLASRENFEDLIHSNLG------------KPVRSKPLS--FDEELKKKVQSRQVKDSSEQQTKITEEKIDNHAKRI
R. monacensis IrR/Munich ..7 HK---NIQEITKDLRKHDPQIPDISKKPLASRENFEDLIHSDLG------------KPVRSKPLS--FDEELKKKVQSRQVKDSSEQQTKITEEKIDNHAKRI
Candidatus R. amblyommii GAT-30V .11 HK---NIQEITKDLRKHDPQIPDTSKKPLASRENFEDLIHSD---
R. aeschlimannii ..7 HK---NIQEITKDLRKHDPQTPDTSKKPLASRENFEDLIHSDLG------------KPVRNKPLA--FDEELKKKVQSRQVKDSFEQQTKSTEEKIDNHAKRI
R. rhipicephali 3-7-female6-CWPP ..7 HK---NIQEITKDLRKHDPQIPDTSKKPLASRENFEDLIHSDLG------------KPVRNKPLA--FDEELKKKVQIRQVKDSFEQQTKSTEEKIDNHAKRI
R. montanensis OSU 85-930 ..7 HK---NIQEITKDLRKHDPQIPDTSKKPLASRENFEDLIHSDLG------------KPVRNKPLS--FDEELKKKVQSRQVKDSFERQTKSTEEKIDNYAKRI
Candidatus R. gravesii .11 HKNIQNIQEITKNLRKHDPQIPDTSKKPLASRENFEDLIHSDLG------------KPVRNKPLS--FDEELKKKVQSRQVKDSFEQQTKSTEEKIDNHAKRI
R. heilongjiangensis 054 ..7 NK---NIQEITKGLRKHDPQIPDTGKKPLASRENFDDLIHSDLG------------QPVRNKPLS--FDEELKKKVQSRQVQDSFEQQTKSTEEKIDNHAKRI
R. japonica ..7 NK---NIQEITKGLRKHDPQIPDTSKKLLASRENFDGLIHSDLG------------QPVRNEPLS--FDEELKQKVQSRQVQDSFEQQTKSTEEKIDNHAKRI
R. honei .11 HK---NIQEITKDLRKHDPQIPDTSKKTLASRENFDDLIHSDLG------------QPVRNKPLS--FDEEVKKKVQSRQIQDSFEQQTKSTEEEIDNHAKRI
R. philipii 364D ... --
R. rickettsii Brazil ..7 HK---NIQEITKDLRKHDPQIPDTSKKPLASRENFDDLIHSDLG------------QPVRNKPLS--FDEELKKKVQSRQVQDFFEPQTKSTEEKIDNHAKRI
R. slovaca D-CWPP ..7 HK---NIQEITKDLRKHDPQIPNASKKPLASRENFDDLIHSDLG------------QPVRNKPLS--FDEELKKKVQSRQVQDFFEQQTKSTEEKIDNHAKRI
R. conorii Malish_7 .11 YK---NIQEITKDLRKHDPQIPDASKKPLASRENFDDLIHSDLG------------QPVRNKPLS--FDEELKKKVQSRQVQDFFEQQTKSTEEKIDNHAKRI
R. parkeri Portsmouth ... --
R. sibirica ... --
R. bellii RML369-C EELKQKQEDLENKKKELEEKKANSNITKEEKWRIVAELEGVTKHLESIKKEHNTINTAEEREKQF-NKSSQSSALKGEPTAADKQQQAAMELIRLRKEQRLQKEAASKAI
R. bellii OSU 85-389 EELKQKQEDLENKKKELEEKKANSNITKEEKWRIVAELEGVTKHLESIKKEHNTINTAEEREKQF-NKSSQSSALKGEPTAADKQQQAAMELIR-LRKQRLQEEAASKAI
R. canadensis McKiel --
R. tamurae ---MKEEKIKQQQA-EAV
R. helvetica ---MKEEKIKQQQA-EAV
R. australis Cutlack EELKQKQKELEDKKKGLEEKKSNSNITREERWKIVAELESINNQFKAIEKVYNTINSAADREKKFDNKNNPASPTKGEPTAADLQQQGALAAIKRMKEEKIKQQQA-EAV
R. akari Hartford EELKQKQKALEDKKKGLEEKKSNSNITREERWKIVAELESINNQFQAIEKEYNIINSASDREKKFDNKNNPSSPTKGAPTAADLQQQGALAAIKRMKEEKIKQQQA-EAA
R. akari Hartford --MVGITAIKRMKEEKIKQQQA-EAA
R. felis URRWXCal2 EELKQKQKELEDKKKGLEEKKANSNITREERWKIVAELESINNQFKAIEKERSNINSASDREKKFDNKNNPSSPTKGEPTAADLQQQGALAAIKRMKEEKIKQQQA-EAT
R. typhi Wilmington ---I
R. prowazekii Madrid_E ---I
R. prowazekii GvV257 ---I
R. prowazekii Chernikova ---I
REIS EELKQKQKELEDRKKGLEEKKLILILLEKKDGKSLQN---
Rickettsia sp. ISO7 EELKQKQKELEDRKKGLEEKKLILILLEKKDGKSLQN---
R. monacensis IrR/Munich EELKQKQKELEDRKKGLEEKKANSNITREERWKIVAELEAINNQFKAIEKEHNTINSASDREKKFDYKNKHLTSTKREPTAADLQQQGALAVIKRMKEEKIKQQQA-EAV
Candidatus R. amblyommii GAT-30V --
R. aeschlimannii EELKQKQKELEERKKGLEEKKANSNITREERWKIAAELKAINNQFKAIEKEHNTINSASDREKKFDNKNKHLTSTKREPTAADLQQQGALAAIKRMKEEKIKQQQA-EVV
R. rhipicephali 3-7-female6-CWPP EELKQKQKELEERKKGLEEKKANSNITREERWKIAAELEAINNQFKAIEKEHNTINSASDREKKFDNKNKHLTSTKREPTAADLQQQGALAAIKRVKEEKIKQQQA-EAV
R. montanensis OSU 85-930 EELKQKQKELEERQKGLEEKKANSNITREARWKIAAELEAINNQFKAIEKEHNTINSASDREKKFDNKNKHLTSTKREPVAADLQQQGALAAIKRMKEEKIKQQQA-EAV
Candidatus R. gravesii EELKQKQKELEERKKGLEEKKANSNITRDERWKIAAELEAINNQFKAIEKEHNTINSASDREKKFDNKNKHLTSTKREPTAADLQQQGALAAIKRMKEEKIQQQQA-EAV
R. heilongjiangensis 054 EELKQKQKELEERKKGLEEKKANSNITREERWKIAAELEAINNQFKAIEKEHNTINSASDREQKFDNKNKYLTSTKREPTAADLQQQGALAAIKRMKEEKIKQQQA-EAV
R. japonica EELKQKQKELEERKKGLEEKKANSNITREERWKIAAELEAINNQFKAIEKEHNTINSASDREQKFDNKNKYLTSTKREPTAADLQQQGALAAIKRMKEEKIKQQQA-EAV
R. honei EELKQKQKELEERKKGLEEKKANSNITREERWKIAAELEAINNQFKAIEKEHNTINSASDREQKFDNKNKHLTATKREPIAADL...........MKKE-IKQQQA-EAV
R. philipii 364D ---MKEEKIKQQQA-EAV
R. rickettsii Brazil EELKQKQKELEERQKGLEEKKANSNITREERWKIAAELEAINNQFKAIEKEHNTINSASDREQKFDNKNKHLTATKREPIAAGL...........MKEEKIKQQQA-EAV
R. slovaca D-CWPP EELKQKQKELEERKKWLEEKKANSNITREERWKIAAELEAINNQFKAIEKEHNTINSASDREQKFDNKNKHLTATKREPIAADL...........MKEEKIKQQQA-EAV
R. conorii Malish_7 EELKQKQKELEERKKWLEEKKANSNITIEERWQIAAELEAINTQLKAIEKEHNTINSASDREQKFDNKNKHLTATKREPIAADL..........................
R. parkeri Portsmouth --
R. sibirica --MGSYKTYERREDKA-AAV

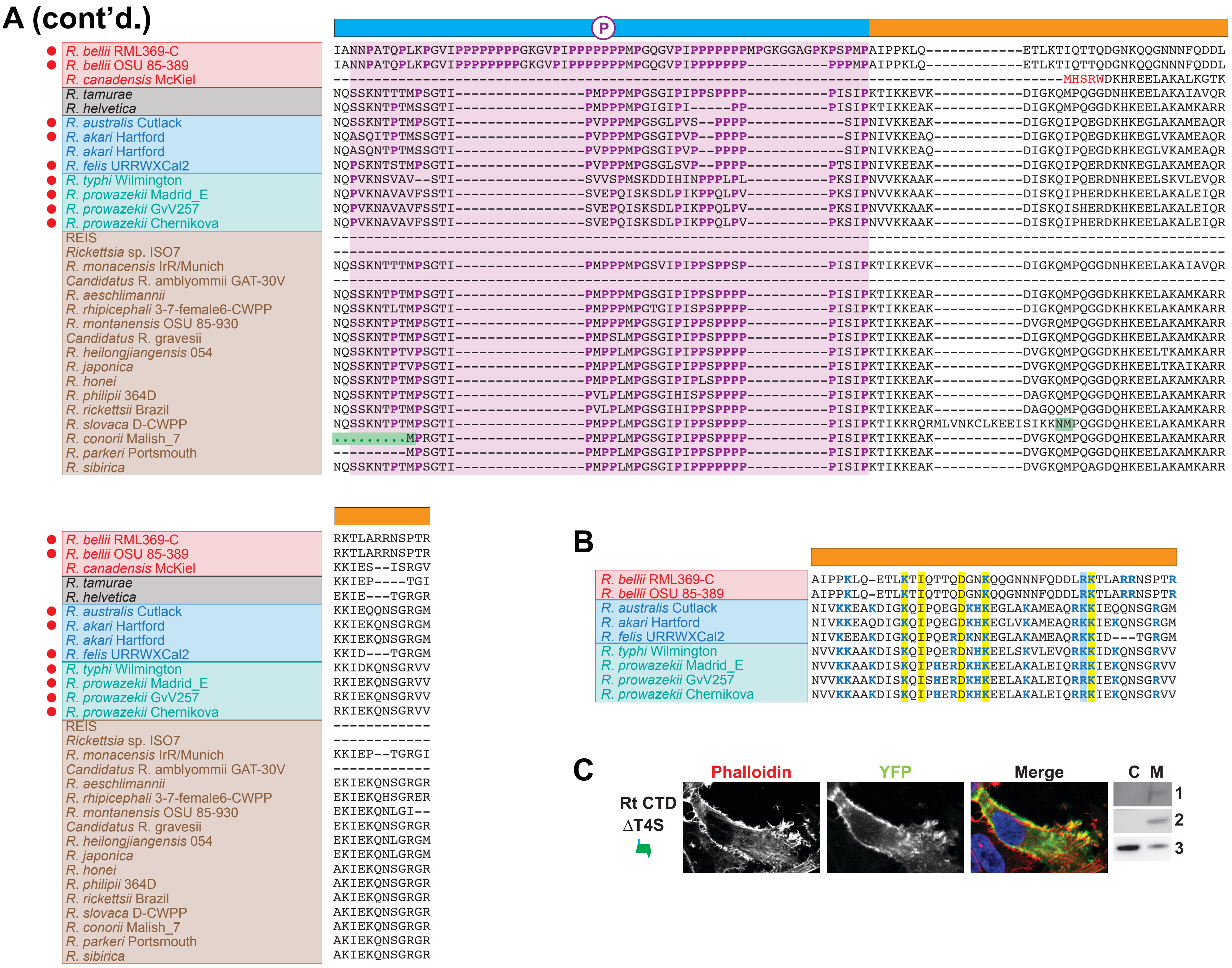
A (cont'd.)
P
R. bellii RML369-C IANNPATQPLKPGVIPPPPPPPPGKGVPIPPPPPPPMPGQGVPIPPPPPPPMPGKGGAGPKPSPMPAIPPKLQ------------ETLKTIQTTQDGNKQQGNNNFQDDL
R. bellii OSU 85-389 IANNPATQPLKPGVIPPPPPPPPGKGVPIPPPPPPPMPGQGVPIPPPPPPP----------PPPMPAIPPKLQ------------ETLKTIQTTQDGNKQQGNNNFQDDL
R. canadensis McKiel ---MHSRWDKHREELAKALKGTK
R. tamurae NQSSKNTTTMPSGTI----------------PMPPPMPGSGIPIPPSPPPP----------PISIPKTIKKEVK-----------DIGKQMPQGGDNHKEELAKAIAVQR
R. helvetica NQSSKNTPTMSSGTI----------------PMPPPMPGIGIPI-----PP----------PISIPKTIKKEAK-----------DIGKQMPQGGDKHKEELAKAMKARR
R. australis Cutlack NQSSKNTPTMPSGTI----------------PVPPPMPGSGLPVS--PPPP------------SIPNIVKKEAK-----------DIGKQIPQEGDKHKEGLAKAMEAQR
R. akari Hartford NQASQITPTMSSGTI----------------PVPPPMPGSGIPVP--PPPP------------SIPNIVKKEAQ-----------DIGKQIPQEGDKHKEGLVKAMEAQR
R. akari Hartford NQASQNTPTMSSGTI----------------PVPPPMPGSGIPVP--PPPP------------SIPNIVKKEAQ-----------DIGKQIPQEGDKHKEGLVKAMEAQR
R. felis URRWXCal2 NQPSKNTSTMPSGTI----------------PVPPPMPGSGLSVP--PPPP----------PTSIPNIVKEEAK-----------DIGKQIPQERDKNKEGLAKAMEAQR
R. typhi Wilmington NQPVKNSVAV--STI----------------SVVSPMSKDDIHINPPPLPL----------PKSIPNVVKKAAK-----------DISKQIPQERDNHKEELSKVLEVQR
R. prowazekii Madrid_E NQPVKNAVAVFSSTI----------------SVEPQISKSDLPIKPPQLPV----------PKSIPNVVKKAAK-----------DISKQIPHERDKHKEELAKALEIQR
R. prowazekii GvV257 NQPVKNAVAVFSSTI----------------SVEPQISKSDLPIKPPQLPV----------PKSIPNVVKKAAK-----------DISKQISHERDKHKEELAKALEIQR
R. prowazekii Chernikova NQPVKNAVAVFSSTI----------------SVEPQISKSDLPIKPPQLPV----------PKSIPNVVKKAAK-----------DISKQIPHERDKHKEELAKALEIQR
REIS --
Rickettsia sp. ISO7 --
R. monacensis IrR/Munich NQSSKNTTTMPSGTI----------------PMPPPMPGSVIPIPPSPPSP----------PISIPKTIKKEVK-----------DIGKQMPQGGDNHKEELAKAIAVQR
Candidatus R. amblyommii GAT-30V --
R. aeschlimannii NQSSKNTPTMPSGTI----------------PMPPPMPGSGIPIPPSPPPP----------PISIPKTIKKEAR-----------DIGKQMPQGGDKHKKELAKAMKARR
R. rhipicephali 3-7-female6-CWPP NQSSKNTLTMPSGTI----------------PMPPPMPGTGIPISPSPPPP----------PISIPKTIKKEAK-----------DIGKQMPQGGDKHKKELAKAMKARR
R. montanensis OSU 85-930 NQSSKNTPTMPSGTI----------------PMPPPMPGSGIPIPPSPPPP----------PISIPKTIKKEAK-----------DVGRQMPQGGDKHKEELAKAMKARR
Candidatus R. gravesii NQSSKNTPTMPSGTI----------------PMPSLMPGSGIPIPPSPPPP----------PISIPKTIKKEAK-----------DIGKQMPQGGDKHKEELAKAMKARR
R. heilongjiangensis 054 NQSSKNTPTVPSGTI----------------PMPPLMPGSGIPIPPSPPPP----------PISIPKTIKKEAK-----------DVGKQMPQGGDKHKEELTKAMKARR
R. japonica NQSSKNTPTVPSGTI----------------PMPPLMPGSGIPIPPSPPPP----------PISIPKTIKKEAK-----------DVGKQMPQGGDKHKEELTKAIKARR
R. honei NQSSKNTPTMPSGTI----------------PMPPLMPGSGIPIPLSPPPP----------PISIPKTIKKEAK-----------DVGKQMPQGGDQRKEELAKAMKARR
R. philipii 364D NQSSKNTPTMPSGTI----------------PVLPLMPGSGIHISPSPPPP----------PISIPKTIKKEAK-----------DAGKQMPQGGDQHKEELAKAMKARR
R. rickettsii Brazil NQSSKNTPTMPSGTI----------------PVLPLMPGSGIHIPPSPPPP----------PISIPKTIKKEAK-----------DAGQQMPQGGDQHKEELAKAMKARR
R. slovaca D-CWPP NQSSKNTPTMPSGTI----------------PMPPLMPGSGIPIPPSPPPP----------PISIPKTIKKRQRMLVNKCLKEEISIKKNMPQGGDQHKEELAKAMKARR
R. conorii Malish_7 ....MPRGTI----------------PMPPLMPGSGIPIPPSPPPP----------PISIPKTIKKEAK-----------DVGKQMPQGGDQHKEELAKAMKARR
R. parkeri Portsmouth ---------MPSGTI----------------PMPPLMPGSGIPIPPSPPPP----------PISIPKTIKKEAK-----------DVGKQMPQGGDQHKEELAKAMKARR
R. sibirica NQSSKNTPTMPSGTI----------------PMPPLMPGSGIPIPPPPPPP----------PISIPKTIKKEAK-----------DVGKQMPQAGDQHKEELAKAMKARR
R. bellii RML369-C RKTLARRNSPTR
R. bellii OSU 85-389 RKTLARRNSPTR
R. canadensis McKiel KKIES--ISRGV
R. tamurae KKIEP----TGI
R. helvetica EKIE---TGRGR
R. australis Cutlack KKIEQQNSGRGM
R. akari Hartford KKIEKQNSGRGM
R. akari Hartford KKIEKQNSGRGM
R. felis URRWXCal2 KKID---TGRGM
R. typhi Wilmington KKIDKQNSGRVV
R. prowazekii Madrid_E RKIEKQNSGRVV
R. prowazekii GvV257 RKIEKQNSGRVV
R. prowazekii Chernikova RKIEKQNSGRVV
REIS ------------
Rickettsia sp. ISO7 ------------
R. monacensis IrR/Munich KKIEP--TGRGI
Candidatus R. amblyommii GAT-30V ------------
R. aeschlimannii EKIEKQNSGRGR
R. rhipicephali 3-7-female6-CWPP EKIEKQHSGRER
R. montanensis OSU 85-930 EKIEKQNLGI--
Candidatus R. gravesii EKIEKQNSGRGR
R. heilongjiangensis 054 EKIEKQNLGRGM
R. japonica EKIEKQNLGRGM
R. honei AKIEKQNSGRGR
R. philipii 364D AKIEKQNSGRGR
R. rickettsii Brazil AKIEKQNSGRGR
R. slovaca D-CWPP AKIEKQNSGRGR
R. conorii Malish_7 AKIEKQNSGRGR
R. parkeri Portsmouth AKIEKQNSGRGR
R. sibirica AKIEKQNSGRGR
B
R. bellii RML369-C AIPPKLQ-ETLKTIQTTQDGNKQQGNNNFQDDLRKTLARRNSPTR
R. bellii OSU 85-389 AIPPKLQ-ETLKTIQTTQDGNKQQGNNNFQDDLRKTLARRNSPTR
R. australis Cutlack NIVKKEAKDIGKQIPQEGDKHKEGLAKAMEAQRKKIEQQNSGRGM
R. akari Hartford NIVKKEAQDIGKQIPQEGDKHKEGLVKAMEAQRKKIEKQNSGRGM
R. felis URRWXCal2 NIVKEEAKDIGKQIPQERDKNKEGLAKAMEAQRKKID---TGRGM
R. typhi Wilmington NVVKKAAKDISKQIPQERDNHKEELSKVLEVQRKKIDKQNSGRVV
R. prowazekii Madrid_E NVVKKAAKDISKQIPHERDKHKEELAKALEIQRRKIEKQNSGRVV
R. prowazekii GvV257 NVVKKAAKDISKQISHERDKHKEELAKALEIQRRKIEKQNSGRVV
R. prowazekii Chernikova NVVKKAAKDISKQIPHERDKHKEELAKALEIQRRKIEKQNSGRVV
C
Rt CTD
ΔT4S
Phalloidin
YFP
Merge
C M
1
2
3

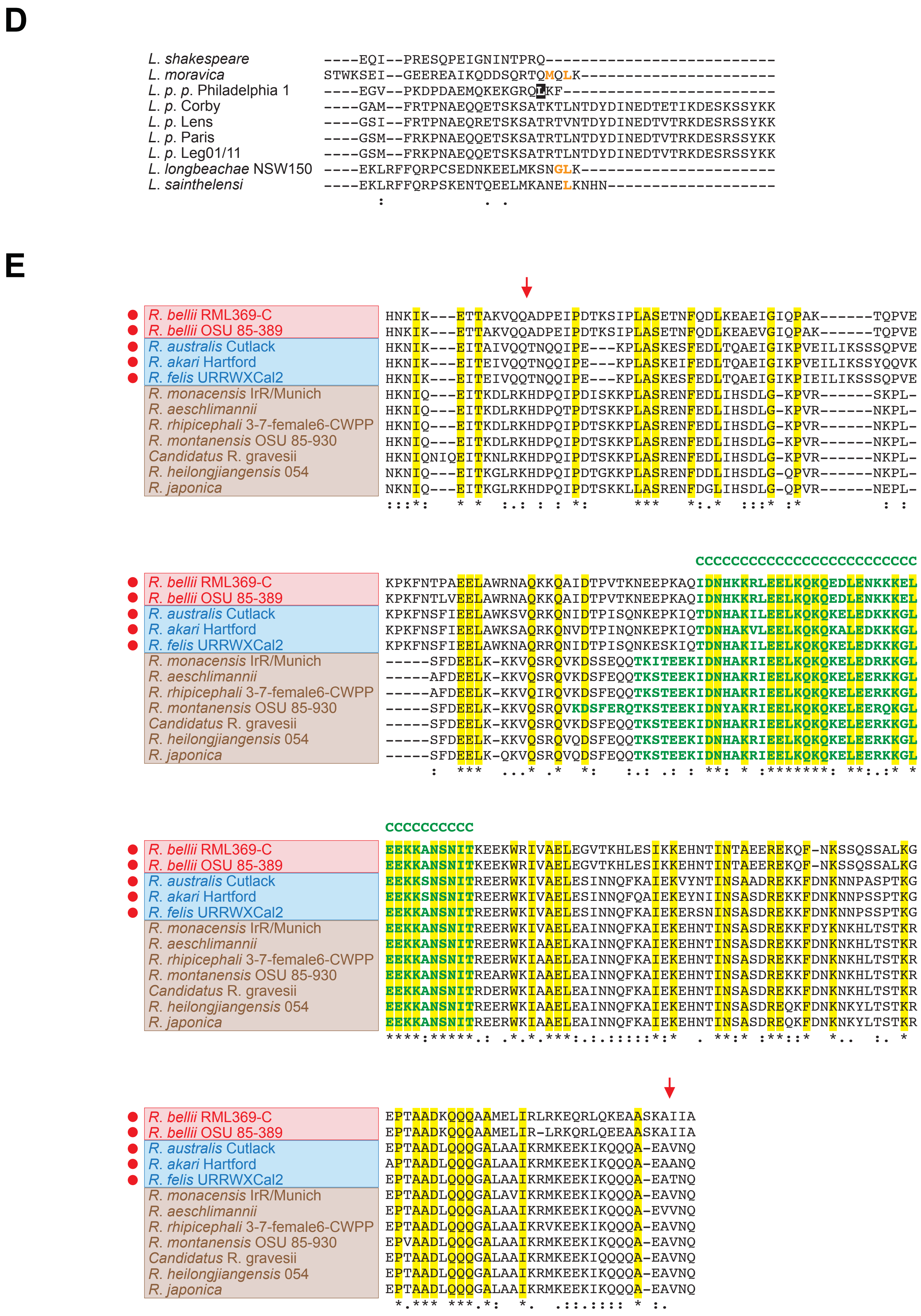
D
L. shakespeare ----EQI--PRESQPEIGNINTPRQ--------------------------
L. moravica STWKSEI--GEEREAIKQDDSQRTQMQLK-----------------------
L. p. p. Philadelphia 1 ----EGV--PKDPDAEMQKEKGRQLKF------------------------
L. p. Corby ----GAM--FRTPNAEQQETSKSATKTLNTDYDINEDTETIKDESKSSYKK
L. p. Lens ----GSI--FRTPNAEQRETSKSATRTVNTDYDINEDTVTRKDESRSSYKK
L. p. Paris ----GSM--FRKPNAEQQETSKSATRTLNTDYDINEDTVTRKDESRSSYKK
L. p. Leg01/11 ----GSM--FRKPNAEQQETSKSATRTLNTDYDINEDTVTRKDESRSSYKK
L. longbeachae NSW150 ----EKLRFFQRPCSEDNKEELMKSNGLK----------------------
L. sainthelensi ----EKLRFFQRPSKENTQEELMKANELKNHN-------------------
E
R. bellii RML369-C HNKIK---ETTAKVQQADPEIPDTKSIPLASETNFQDLKEAEIGIQPAK------TQPVE
R. bellii OSU 85-389 HNKIK---ETTAKVQQADPEIPDTKSIPLASETNFQDLKEAEVGIQPAK------TQPVE
R. australis Cutlack HKNIK---EITAIVQQTNQQIPE---KPLASKESFEDLTQAEIGIKPVEILIKSSSQPVE
R. akari Hartford HKNIK---EITEIVQQTNQQIPE---KPLASKEIFEDLTQAEIGIKPVEILIKSSYQQVK
R. felis URRWXCal2 HKNIK---EITEIVQQTNQQIPE---KPLASKESFEDLTQAEIGIKPIEILIKSSSQPVE
R. monacensis IrR/Munich HKNIQ---EITKDLRKHDPQIPDISKKPLASRENFEDLIHSDLG-KPVR------SKPL-
R. aeschlimannii HKNIQ---EITKDLRKHDPQTPDTSKKPLASRENFEDLIHSDLG-KPVR------NKPL-
R. rhipicephali 3-7-female6-CWPP HKNIQ---EITKDLRKHDPQIPDTSKKPLASRENFEDLIHSDLG-KPVR------NKPL-
R. montanensis OSU 85-930 HKNIQ---EITKDLRKHDPQIPDTSKKPLASRENFEDLIHSDLG-KPVR------NKPL-
Candidatus R. gravesii HKNIQNIQEITKNLRKHDPQIPDTSKKPLASRENFEDLIHSDLG-KPVR------NKPL-
R. heilongjiangensis 054 NKNIQ---EITKGLRKHDPQIPDTGKKPLASRENFDDLIHSDLG-QPVR------NKPL-
R. japonica NKNIQ---EITKGLRKHDPQIPDTSKKLLASRENFDGLIHSDLG-QPVR------NEPL-
CCCCCCCCCCCCCCCCCCCCCCCCCC
R. bellii RML369-C KPKFNTPAEELAWRNAQKKQAIDTPVTKNEEPKAQIDNHKKRLEELKQKQEDLENKKKEL
R. bellii OSU 85-389 KPKFNTLVEELAWRNAQKKQAIDTPVTKNEEPKAQIDNHKKRLEELKQKQEDLENKKKEL
R. australis Cutlack KPKFNSFIEELAWKSVQRKQNIDTPISQNKEPKIQTDNHAKILEELKQKQKELEDKKKGL
R. akari Hartford KPKFNSFIEELAWKSAQRKQNVDTPINQNKEPKIQTDNHAKVLEELKQKQKALEDKKKGL
R. felis URRWXCal2 KPKFNSFIEELAWKNAQRRQNIDTPISQNKESKIQTDNHAKILEELKQKQKELEDKKKGL
R. monacensis IrR/Munich -----SFDEELK-KKVQSRQVKDSSEQQTKITEEKIDNHAKRIEELKQKQKELEDRKKGL
R. aeschlimannii -----AFDEELK-KKVQSRQVKDSFEQQTKSTEEKIDNHAKRIEELKQKQKELEERKKGL
R. rhipicephali 3-7-female6-CWPP -----AFDEELK-KKVQIRQVKDSFEQQTKSTEEKIDNHAKRIEELKQKQKELEERKKGL
R. montanensis OSU 85-930 -----SFDEELK-KKVQSRQVKDSFERQTKSTEEKIDNYAKRIEELKQKQKELEERQKGL
Candidatus R. gravesii -----SFDEELK-KKVQSRQVKDSFEQQTKSTEEKIDNHAKRIEELKQKQKELEERKKGL
R. heilongjiangensis 054 -----SFDEELK-KKVQSRQVQDSFEQQTKSTEEKIDNHAKRIEELKQKQKELEERKKGL
R. japonica -----SFDEELK-QKVQSRQVQDSFEQQTKSTEEKIDNHAKRIEELKQKQKELEERKKGL
CCCCCCCCCC
R. bellii RML369-C EEKKANSNITKEEKWRIVAELEGVTKHLESIKKEHNTINTAEEREKQF-NKSSQSSALKG
R. bellii OSU 85-389 EEKKANSNITKEEKWRIVAELEGVTKHLESIKKEHNTINTAEEREKQF-NKSSQSSALKG
R. australis Cutlack EEKKSNSNITREERWKIVAELESINNQFKAIEKVYNTINSAADREKKFDNKNNPASPTKG
R. akari Hartford EEKKSNSNITREERWKIVAELESINNQFQAIEKEYNIINSASDREKKFDNKNNPSSPTKG
R. felis URRWXCal2 EEKKANSNITREERWKIVAELESINNQFKAIEKERSNINSASDREKKFDNKNNPSSPTKG
R. monacensis IrR/Munich EEKKANSNITREERWKIVAELEAINNQFKAIEKEHNTINSASDREKKFDYKNKHLTSTKR
R. aeschlimannii EEKKANSNITREERWKIAAELKAINNQFKAIEKEHNTINSASDREKKFDNKNKHLTSTKR
R. rhipicephali 3-7-female6-CWPP EEKKANSNITREERWKIAAELEAINNQFKAIEKEHNTINSASDREKKFDNKNKHLTSTKR
R. montanensis OSU 85-930 EEKKANSNITREARWKIAAELEAINNQFKAIEKEHNTINSASDREKKFDNKNKHLTSTKR
Candidatus R. gravesii EEKKANSNITRDERWKIAAELEAINNQFKAIEKEHNTINSASDREKKFDNKNKHLTSTKR
R. heilongjiangensis 054 EEKKANSNITREERWKIAAELEAINNQFKAIEKEHNTINSASDREQKFDNKNKYLTSTKR
R. japonica EEKKANSNITREERWKIAAELEAINNQFKAIEKEHNTINSASDREQKFDNKNKYLTSTKR
R. bellii RML369-C EPTAADKQQQAAMELIRLRKEQRLQKEAASKAIIA
R. bellii OSU 85-389 EPTAADKQQQAAMELIR-LRKQRLQEEAASKAIIA
R. australis Cutlack EPTAADLQQQGALAAIKRMKEEKIKQQQA-EAVNQ
R. akari Hartford APTAADLQQQGALAAIKRMKEEKIKQQQA-EAANQ
R. felis URRWXCal2 EPTAADLQQQGALAAIKRMKEEKIKQQQA-EATNQ
R. monacensis IrR/Munich EPTAADLQQQGALAVIKRMKEEKIKQQQA-EAVNQ
R. aeschlimannii EPTAADLQQQGALAAIKRMKEEKIKQQQA-EVVNQ
R. rhipicephali 3-7-female6-CWPP EPTAADLQQQGALAAIKRVKEEKIKQQQA-EAVNQ
R. montanensis OSU 85-930 EPVAADLQQQGALAAIKRMKEEKIKQQQA-EAVNQ
Candidatus R. gravesii EPTAADLQQQGALAAIKRMKEEKIQQQQA-EAVNQ
R. heilongjiangensis 054 EPTAADLQQQGALAAIKRMKEEKIKQQQA-EAVNQ
R. japonica EPTAADLQQQGALAAIKRMKEEKIKQQQA-EAVNQ

F

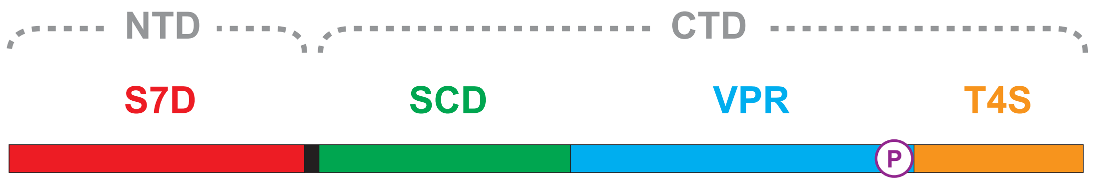
NTD
CTD
S7D
SCD
VPR
T4S
P

G

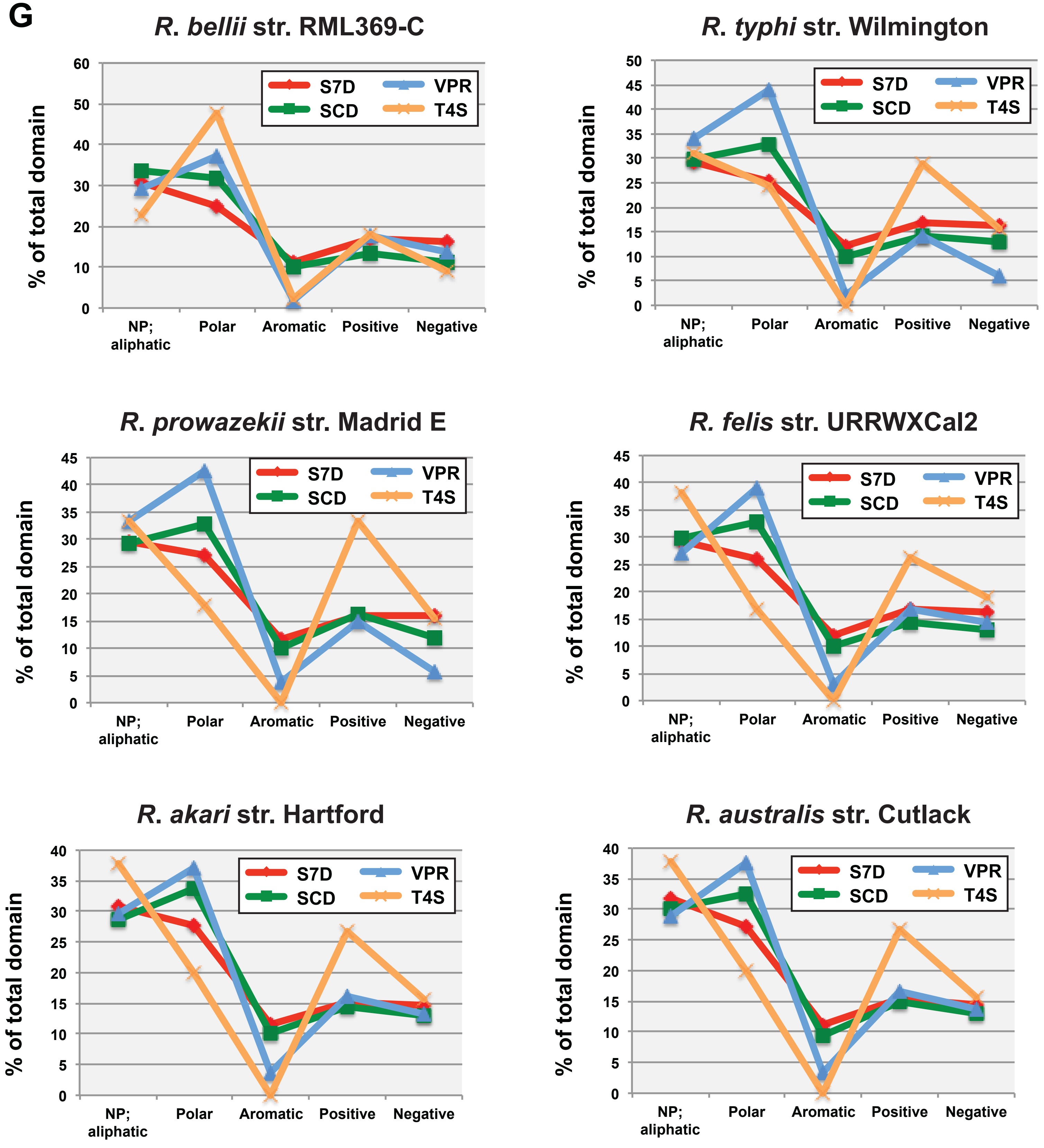
R. bellii str. RML369-C
R. typhi str. Wilmington
R. prowazekii str. Madrid E
R. felis str. URRWXCal2
R. akari str. Hartford
R. australis str. Cutlack
% of total domain
S7D
SCD
VPR
T4S
NP; aliphatic
Polar
Aromatic
Positive
Negative
