# Supplementary material for: Which Way In? The RalF Arf-GEF Orchestrates Rickettsia Host Cell Invasion
Source: PLoS Pathog. 2015 Aug 20;11(8):e1005115. doi: 10.1371/journal.ppat.1005115 (PMC4546372; doi:10.1371/journal.ppat.1005115)
Supplement: S5 Fig — (PDF) [file ppat.1005115.s005.pdf]

S5 Fig. Genome synteny analysis across the *ralF*$_R$ loci from select *Rickettsia* genomes. For select *Rickettsia* genomes, gene neighborhood models were constructed using the Kyoto Encyclopedia of Genes and Genomes (KEGG) database [1], with manual adjustment to gene annotations. Genome coordinates are provided or each species, except for *R*. *bellii*, for which four separate loci are shown that contain genes found in the single *ralF* loci of all other *Rickettsia* species. The genome coordinates for these separate loci are as follows: a, 714015-714959; b, 955271-959620; c, 1037705-1038928; d, 1082690-1086679. These separate loci reflect the substantial gene rearrangement in *R*. *bellii* genomes relative to all other *Rickettsia* species [2]. (A) *ralF* gene neighborhood models for ten *Rickettsia* species. The six species at top contain genes encoding full-length RalF proteins (noted with 'Sec7' on the *ralF* gene model). The remaining four species contain *ralF* genes that lack the regions encoding the S7D and SCD. (B) The genome of *R*. *akari* is unique in that it contains a second *ralF* locus, which is found within a highly pseudogenized region that flanks a cluster of *rvh* (Rickettsiales *vir* homolog) type IV secretion systems genes.

References

1. Ogata H, Goto S, Sato K, Fujibuchi W, Bono H, Kanehisa M. KEGG: Kyoto encyclopedia of genes and genomes. Nucleic Acids Research. 1999. pp. 29–34. doi:10.1093/nar/27.1.29

2. Gillespie JJ, Williams K, Shukla M, Snyder EE, Nordberg EK, Ceraul SM, et al. Rickettsia phylogenomics: Unwinding the intricacies of obligate intracellular life. PLoS One. 2008;3. doi:10.1371/journal.pone.0002018

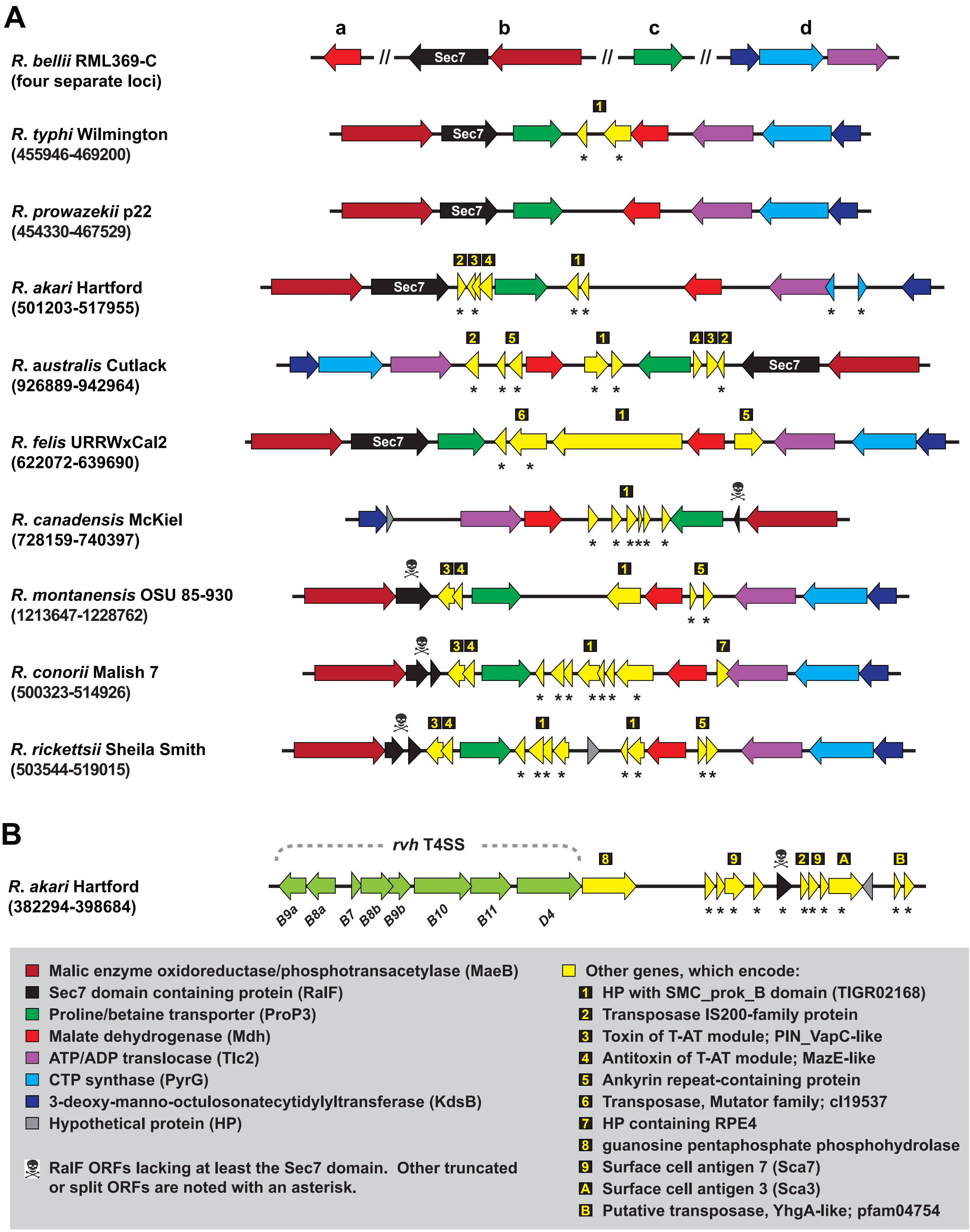

A
a
b
c
d
R. bellii RML369-C
(four separate loci)
Sec7
R. typhi Wilmington
(455946-469200)
R. prowazekii p22
(454330-467529)
R. akari Hartford
(501203-517955)
R. australis Cutlack
(926889-942964)
R. felis URRWxCal2
(622072-639690)
R. canadensis McKiel
(728159-740397)
R. montanensis OSU 85-930
(1213647-1228762)
R. conorii Malish 7
(500323-514926)
R. rickettsii Sheila Smith
(503544-519015)
B
rvh T4SS
R. akari Hartford
(382294-398684)
B9a
B8a
B7
B8b
B9b
B10
B11
D4
Malic enzyme oxidoreductase/phosphotransacetylase (MaeB)
Sec7 domain containing protein (RalF)
Proline/betaine transporter (ProP3)
Malate dehydrogenase (Mdh)
ATP/ADP translocase (Tlc2)
CTP synthase (PyrG)
3-deoxy-manno-octulosonatecytidylyltransferase (KdsB)
Hypothetical protein (HP)
RalF ORFs lacking at least the Sec7 domain. Other truncated or split ORFs are noted with an asterisk.
Other genes, which encode:
1 HP with SMC_prok_B domain (TIGR02168)
2 Transposase IS200-family protein
3 Toxin of T-AT module; PIN_VapC-like
4 Antitoxin of T-AT module; MazE-like
5 Ankyrin repeat-containing protein
6 Transposase, Mutator family; cl19537
7 HP containing RPE4
8 guanosine pentaphosphate phosphohydrolase
9 Surface cell antigen 7 (Sca7)
A Surface cell antigen 3 (Sca3)
B Putative transposase, YhgA-like; pfam04754
