# Supplementary material for: Which Way In? The RalF Arf-GEF Orchestrates Rickettsia Host Cell Invasion
Source: PLoS Pathog. 2015 Aug 20;11(8):e1005115. doi: 10.1371/journal.ppat.1005115 (PMC4546372; doi:10.1371/journal.ppat.1005115)
Supplement: S6 Fig — (PDF) [file ppat.1005115.s006.pdf]

S6 Fig. Densitometry analysis of membrane fractionation. Image J was used to perform densitometry analysis of anti-GFP and anti-GAPDH protein immunoblots (Fig 3). Arbitrary units for GFP intensity was normalized to GAPDH intensity and the percent of cytoplasmic and membrane association for each protein was calculated. Mean ± SEM of two independent analyses are plotted.

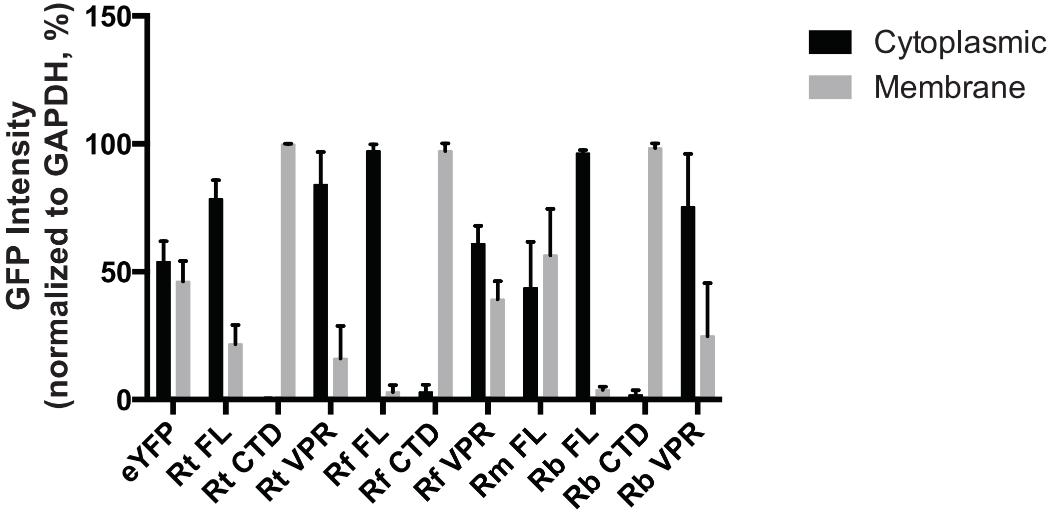

GFP Intensity
(normalized to GAPDH, %)
150
100
50
0
eYFP
Rt FL
Rt CTD
Rt VPR
Rf FL
Rf CTD
Rf VPR
Rm FL
Rb FL
Rb CTD
Rb VPR
Cytoplasmic
Membrane
