# Supplementary material for: Which Way In? The RalF Arf-GEF Orchestrates Rickettsia Host Cell Invasion
Source: PLoS Pathog. 2015 Aug 20;11(8):e1005115. doi: 10.1371/journal.ppat.1005115 (PMC4546372; doi:10.1371/journal.ppat.1005115)
Supplement: S7 Fig — (PDF) [file ppat.1005115.s007.pdf]

S7 Fig. Subcellular localization of rickettsial RalF proteins to Golgi apparatus.

HeLa cells expressing YFP tagged RalF proteins (green, described in Fig 2B) were fixed and the Golgi apparatus detected with mouse anti-GM130 and Alexa Fluor 594 anti-mouse antibodies. DAPI (blue) is shown in the merged image. (Scale bar: 10 μm)

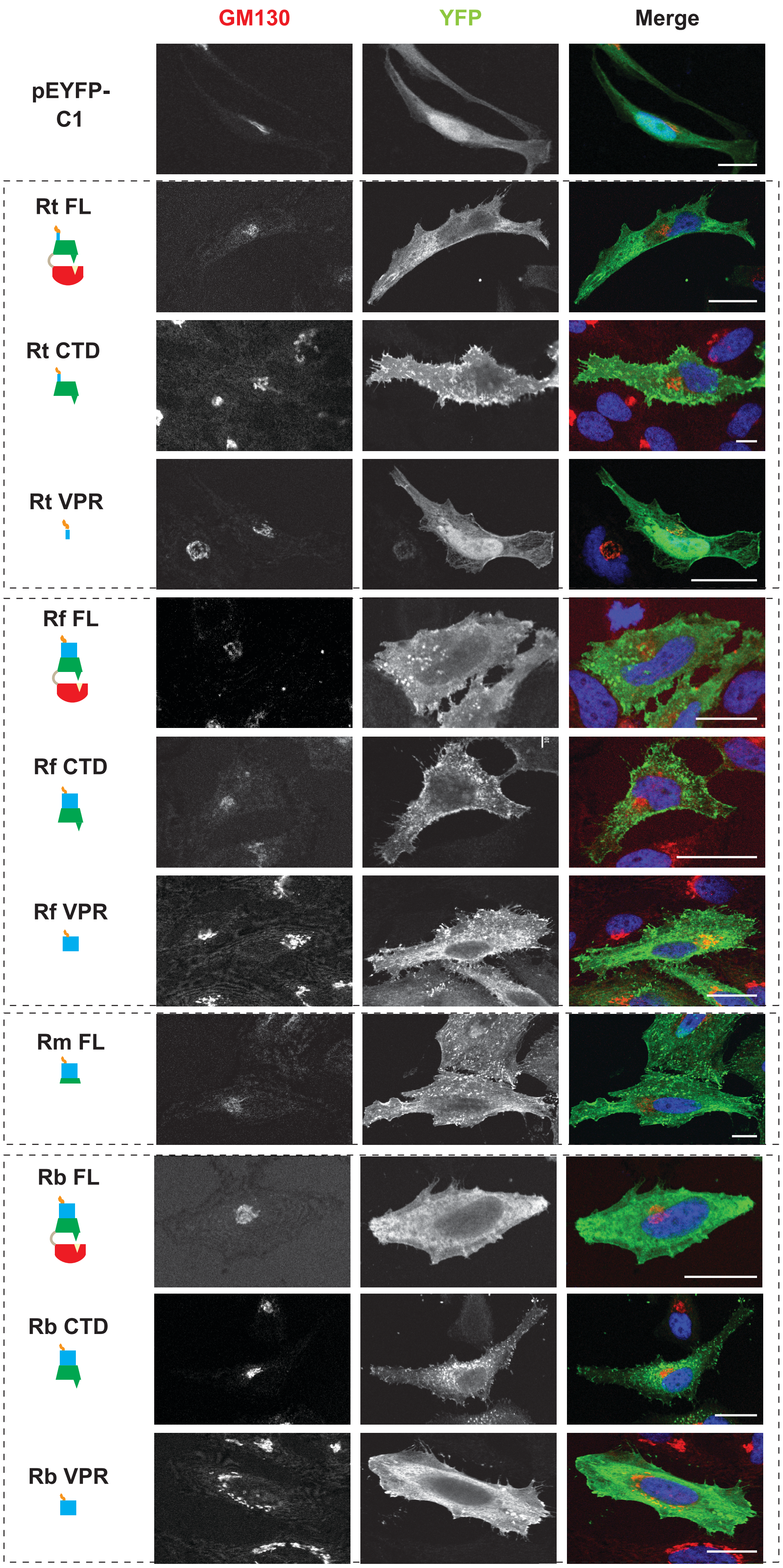

GM130
YFP
Merge
pEYFP-C1
Rt FL
Rt CTD
Rt VPR
Rf FL
Rf CTD
Rf VPR
Rm FL
Rb FL
Rb CTD
Rb VPR
