# Supplementary material for: Which Way In? The RalF Arf-GEF Orchestrates Rickettsia Host Cell Invasion
Source: PLoS Pathog. 2015 Aug 20;11(8):e1005115. doi: 10.1371/journal.ppat.1005115 (PMC4546372; doi:10.1371/journal.ppat.1005115)
Supplement: S8 Fig — (PDF) [file ppat.1005115.s008.pdf]

S8 Fig. Quantification of rickettsial RalF proteins co-localization with plasma membrane, endoplasmic reticulum and Golgi apparatus. Pearson's correlation coefficients for measuring co-localization of RalF proteins with WGA (A), PDI (B), or GM130 (C), markers of the plasma membrane, endoplasmic reticulum and Golgi apparatus, respectively. Five to ten cells per condition from two independent experiments were analyzed using the CoLoc2 plugin in the ImageJ software program and mean Pearson's correlation coefficients ± SEM is plotted. (* $P < 0.05$ compared with eYFP control, two-tailed Student's t-test)

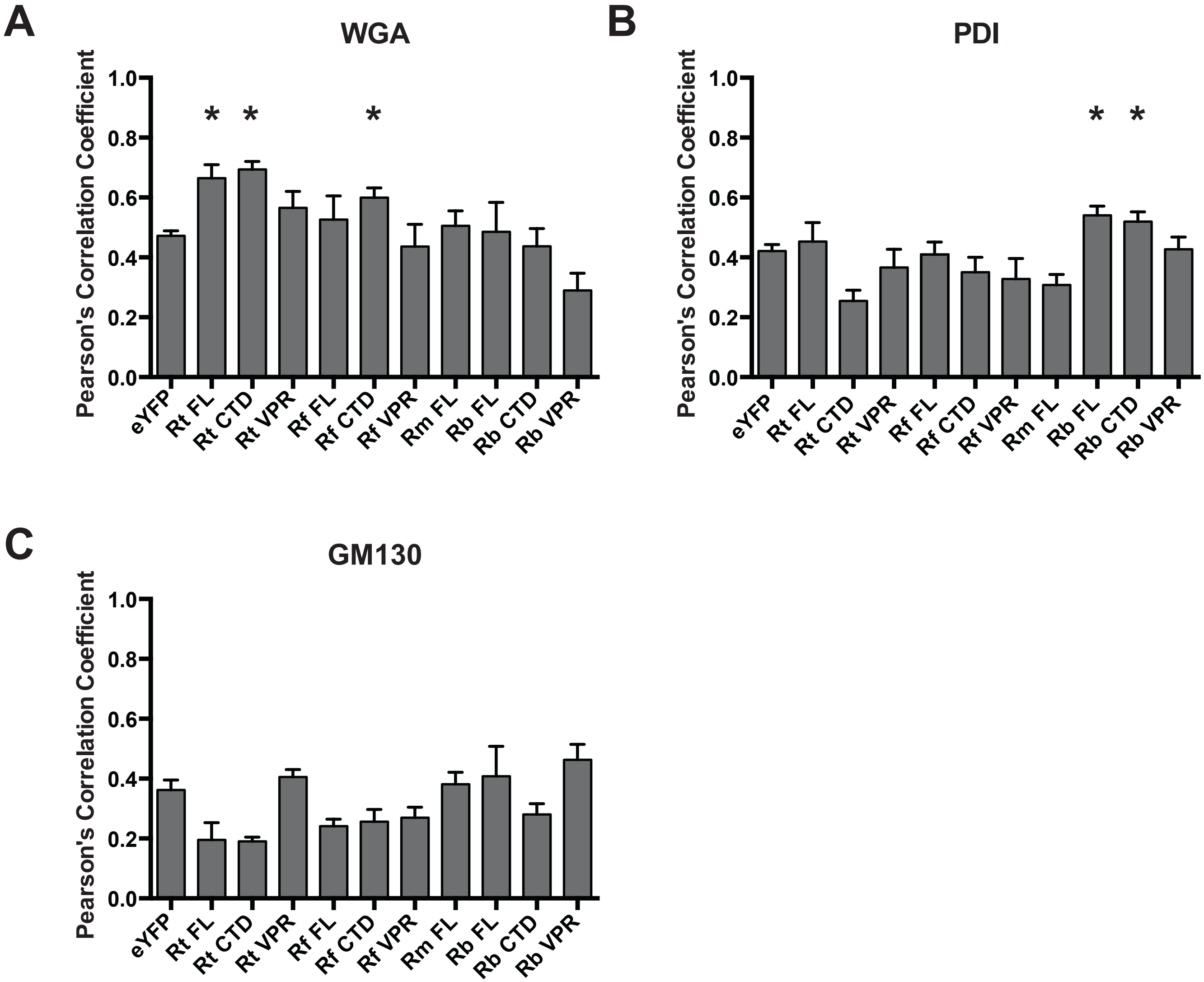
A
WGA
B
PDI
C
GM130
Pearson's Correlation Coefficient
1.0
0.8
0.6
0.4
0.2
0.0
*
eYFP
Rt FL
Rt CTD
Rt VPR
Rf FL
Rf CTD
Rf VPR
Rm FL
Rb FL
Rb CTD
Rb VPR
