# Supplementary material for: Which Way In? The RalF Arf-GEF Orchestrates Rickettsia Host Cell Invasion
Source: PLoS Pathog. 2015 Aug 20;11(8):e1005115. doi: 10.1371/journal.ppat.1005115 (PMC4546372; doi:10.1371/journal.ppat.1005115)
Supplement: S9 Fig — (PDF) [file ppat.1005115.s009.pdf]

S9 Fig. Arf1 does not co-localize with $RalF_R$. HeLa cells co-expressing EYFP, EYFP-$RalF_{RtFL}$ or EYFP-$RalF_{RbFL}$ and mRFP-Arf1 were fixed with 4% para-formaldehyde. Nuclei were stained with DAPI (blue). (Scale bar: 10 μm)

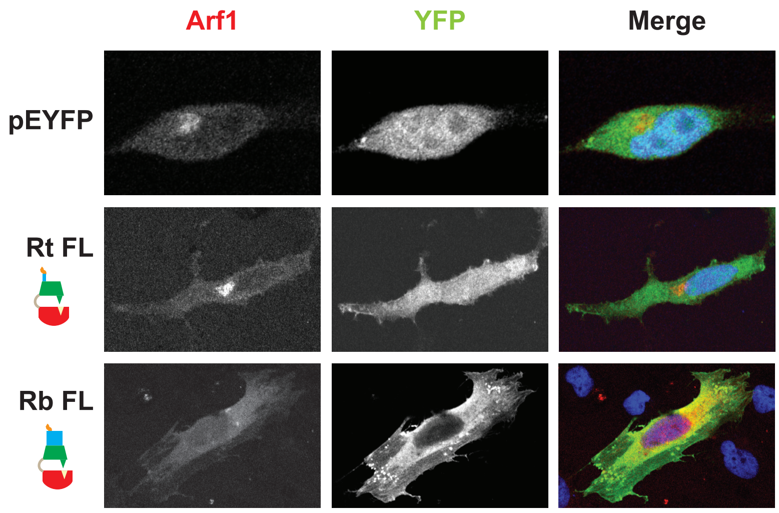
Arf1
YFP
Merge
pEYFP
Rt FL
Rb FL
