# Supplementary material for: Which Way In? The RalF Arf-GEF Orchestrates Rickettsia Host Cell Invasion
Source: PLoS Pathog. 2015 Aug 20;11(8):e1005115. doi: 10.1371/journal.ppat.1005115 (PMC4546372; doi:10.1371/journal.ppat.1005115)
Supplement: S1 Table — (PDF) [file ppat.1005115.s010.pdf]

Table S1. Primers used for this study.

Construct	Forward Primer (5'-3')	Reverse Primer (5'-3')
pTRG-RalF$_{RtFL}$	GCGGCCGCCATGGATTCATTACTAAAAAACG	CTCGAGTTATACTACTCTCCCTGAATTTTGC
pTRG-RalF$_{Rt\Delta T4S}$	GCGGCCGCCATGGATTCATTACTAAAAAACG	CTCGAGTGGAATTGATTTAGGAAGTGGTAA
pBT- coRvhD4	GCGGCCGCCATGGAATGGCATAAAATCCTG	CTCGAGTTCATTGTTTTCAATTGCGGTG
pTrcHisA-RalF	GGATCCATGGATTCATTACTAAAAAACGAA	GAATTCTTATACTACTCTCCCTGAATTTTG
pEYFP-RalF$_{RtFL}$	GAATTCTATGGATTCATTACTAAAAAACG	GGATCCTTATACTACTCTCCCTGAATTTTGCTTGTC
pEYFP-RalF$_{RtCTD}$	GAATTCTCATAAAAATATTAAAACAGAAGTTGTATATCA AATAAATC	GGATCCTTATACTACTCTCCCTGAATTTTGCTTGTC
pEYFP-RalF$_{RtVPR}$	GAATTCTCATAAAAATATTAAAACAGAAGTTGTATATCA AATAAATC	GGATCCTTATACTACTCTCCCTGAATTTTGCTTGTC
pEYFP-RalF$_{RtCTD\Delta T4S}$	GAATTCTCATAAAAATATTAAAACAGAAGTTGTATATCA AATAAATC	GGATCCTTATGGAATTGATTTAGGAAGTGGTAATGG
pEYFP-RalF$_{RfFL}$	TCGAATTCTATGGACTCATTAG	GGATCCCTACATTCCTCTACC
pEYFP-RalF$_{RfCTD}$	TCGAATTCTCCTGGTTATGAGATAG	GGATCCCTACATTCCTCTACC
pEYFP-RalF$_{RfVPR}$	GAATTCTCATAAAAATATTAAAGAAATTACAGAAATTGT ACAGCAAAC	GGATCCCTACATTCCTCTACC
pEYFP-RalF$_{RmFL}$	TCGAATTCTATGGCGCCGATTATAG	GGATCCTCATATTCCTAAGTTTTGC
pEYFP-RalF$_{RbFL}$	CTCAAGCTTCGAATTCTATGGATCCGTTAATAAAAAGAG AAGTAATAAGTTCCT	TAGATCCGGTGGATCCCTAACGTGTTGGGCTATTTCTC CT
pEYFP-RalF$_{RbCTD}$	GAATTCTACTGGTTATGAAATAGCC	GGATCCCTAACGTGTTGGGCTATTTC
pEYFP-RalF$_{RbVPR}$	GAATTCTCATAATAAAATAAAAGAAACTAC	GGATCCCTAACGTGTTGGGCTATTTC
